# High Disease Burden and Oral Corticosteroid Use in Patients with Hypereosinophilic Syndrome and Eosinophilic Granulomatosis with Polyangiitis: Country-Level Insights into Real-World Management in Europe

**DOI:** 10.3390/jcm14124309

**Published:** 2025-06-17

**Authors:** Jeremiah Hwee, Lynn Huynh, Thanai Pongdee, Marc E. Rothenberg, Rafael Alfonso-Cristancho, Wilson da Costa Junior, Mei Sheng Duh

**Affiliations:** 1 Epidemiology, GSK, 100 Milverton Drive, Mississauga, ON L5R 4H1, Canada; 2 Analysis Group, Inc., Boston, MA 02199, USA; 3 Division of Allergic Diseases, Mayo Clinic, Rochester, MN 55905, USA; 4 Department of Pediatrics, Division of Allergy and Immunology, Cincinnati Children’s Hospital Medical Center, Cincinnati, OH 45229-3026, USA; 5 School of Medicine, University of Cincinnati, Cincinnati, OH 45267-0555, USA; 6 Global Real-World Evidence and Health Outcomes Research, GSK, Collegeville, PA 19426, USA

**Keywords:** oral corticosteroids, biologic therapies, treatment variability, eosinophilic granulomatosis with polyangiitis (EGPA), hypereosinophilic syndrome (HES), healthcare resource utilization, management strategies

## Abstract

**Objectives:** To analyze variations in patient characteristics, treatment patterns, clinical manifestations, clinical outcomes (i.e., response, flares and flare-free survival in HES; remission, relapses and relapse-free survival in EGPA; and overall survival), and healthcare resource utilization (HCRU) in patients with hypereosinophilic syndrome (HES) and eosinophilic granulomatosis with polyangiitis (EGPA) across five European countries. **Methods:** In two parallel, retrospective, non-interventional, longitudinal chart review studies (GSK ID: 214661 [EGPA] and 214657 [HES]), physicians collected data of patients they treated in France, Germany, Italy, Spain, and the UK from January 2015 to December 2019, with follow-up until August 2021. Country-level results are presented for each study; HES and EGPA data were pooled in a post hoc exploratory analysis. **Results:** Per-country, 22–26 HES- and 38–45 EGPA-treating physicians collected data from 52–62 (total 280) patients with HES and 80–85 (total 407) with EGPA. Patient sex and age at diagnosis differed across countries. Pooled HES/EGPA data revealed high oral corticosteroid (OCS) use in all countries (94.9% of patients; median [IQR] duration 20.7 [9.0, 33.8] months); immunosuppressive treatments and biologics use varied between countries (43.7–61.5% and 25.6–59.8%, respectively). The most frequent clinical manifestations were constitutional (51.6–78.8%) and lung (43.5–55.8%) in HES, and lung (41.3–67.9) and ENT (43.8–61.2) in EGPA. Pooled HCRU data showed country-level variation; 70.7–91.2% of patients had disease-related outpatient visits and 36.1–52.6% had ER visits or hospitalizations. **Conclusions:** Results demonstrate substantial disease burden, including high HCRU and extensive OCS use among patients with HES and EGPA in five European countries. The findings highlight the need for improved treatment strategies such as optimizing use of biologics to mitigate the reliance on corticosteroids.

## 1. Introduction

Hypereosinophilic syndrome (HES) and eosinophilic granulomatosis with polyangiitis (EGPA) are rare systemic inflammatory diseases with substantial overlap in clinical presentation [1]. Both are characterized by elevated blood eosinophil counts (persistently > 1500 cells/µL in HES and ≥1000 cells/µL in EGPA) and eosinophilic infiltration in tissues, leading to tissue and end-organ damage [2,3,4,5,6]. EGPA differs from HES by the presence of small-to-medium vessel vasculitis [1,2]. In the typical disease course of EGPA, clinical features such as asthma, chronic rhinosinusitis, and eosinophilia typically precede the occurrence of vasculitis, with asthma present in over 90% of cases [1,2,7,8,9]. Both diseases have a heterogeneous presentation, commonly involve multiple organ systems, and share many overlapping symptoms [1,4,7,9,10].

For both HES and EGPA, treatment relies heavily on oral corticosteroids (OCS), with immunosuppressive/cytotoxic therapies used as add-on therapies to improve disease control or for OCS sparing [1,2,3,11,12,13]. Both OCS and immunosuppressive/cytotoxic therapies are associated with substantial adverse effects [1,11,14,15]. Patients with EGPA and HES commonly depend on long-term OCS use or require repeated courses to manage relapses or flare episodes [3,11], increasing the risk of related toxicities [16]. Recently, biologics have been recommended for EGPA, depending on disease severity and activity state, and for certain subtypes of HES, based on their effectiveness and OCS-sparing benefits [1,3,11].

To date, a limited number of published studies have demonstrated that HES and EGPA are associated with a high burden of disease with patients requiring frequent medical visits and hospitalizations [17,18,19,20,21]. Two of these studies reported the overall results from our European longitudinal chart review studies assessing the burden of illness for each condition in five European countries: France, Germany, Italy, Spain, and the UK [17,21]. Despite treatment, patients with HES continue to experience severe clinical manifestations and flares, requiring numerous outpatient visits and hospital admissions [17]. Similarly, many patients with EGPA require inpatient care and ongoing treatment [20]. Due to varied disease manifestations, patients with these diseases often require care by physicians from multiple specialties [2]. Additionally, given the rarity and complexity of diagnosis and low awareness of HES and EGPA, diagnosis is often delayed [22,23].

Herein we expand on our prior publications [17,21], to better understand the variations in patient characteristics, treatment patterns, clinical outcomes, and healthcare resource utilization (HCRU) associated with HES and EGPA in real-world settings at the country level. Additionally, a post hoc pooled analysis of combined data for patients with HES and those with EGPA was performed to identify key trends and core management practices for these diseases across the five countries.

## 2. Materials and Methods

### 2.1. Study Design

**Figure 1:**
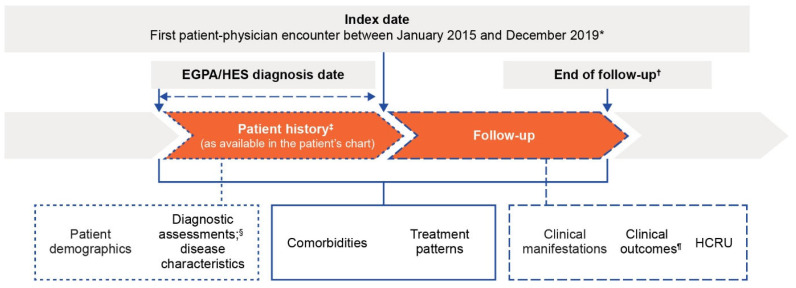
Study design of the HES (214657) and EGPA (214661) retrospective chart review studies. * Patient may have been previously diagnosed with EGPA or HES or was newly diagnosed at the first encounter. ^†^ Earliest date of death, loss to follow-up or date of chart abstraction. ^‡^ For patients diagnosed with EGPA/HES before the index date, data from diagnosis to index were collected from the patient’s chart. Patient demographics and baseline clinical characteristics were identified. ^§^ Includes blood eosinophil counts. ^¶^ Response and remission were assessed from diagnosis date to the EOF. EGPA, eosinophilic granulomatosis with polyangiitis; EOF, end of follow-up; HCRU, healthcare resource utilization; HES, hypereosinophilic syndrome.

This analysis reports real-world country-level data (France, Germany, Italy, Spain, and the UK) from two parallel, retrospective, non-interventional, longitudinal, physician panel-based chart review studies of patients diagnosed with EGPA (study ID 214661) or HES (study ID 214657) [17,21]. Both study designs were similar, and have been published previously (Figure 1) [17,21]. The studies aimed to recruit equal numbers of treating physicians from each country. The index date was the first physician encounter on or after diagnosis of HES or EGPA diagnosis between January 2015 and December 2019. The end date of chart abstraction was August 2021. Data were collected from the index date until the end of follow-up (EOF), which was defined as the earliest date of death, loss to follow-up, or date of chart abstraction.

### 2.2. Patient and Physician Eligibility Criteria

Physicians were recruited from the targeted specialties of allergy, immunology, pulmonology, rheumatology, hematology (HES only), and internal medicine (HES only) from each participating country. Eligible physicians had access to medical records of at least one patient with HES or EGPA and served as the primary healthcare provider for those patients.

Patients aged ≥ 6 years with physician-confirmed diagnosis of HES, without an identifiable non-hematologic secondary cause, and patients aged ≥ 12 years with physician-confirmed diagnosis of EGPA were included. At least 1 year of follow-up data from index to EOF was required to be accessible to the physician, except where follow-up ended due to death.

### 2.3. Data Source and Collection

Anonymized longitudinal patient-level data were obtained from demographically and geographically diverse, nationally representative populations, using standardized electronic case report forms. Quality control measures included an automated consistency check and identification of physicians who had short response times or convenient response selection, who were then excluded from the analysis. The eCRF included a screening survey which confirmed eligibility of the participating physician. Completing physicians were blind to the identity of the study sponsor and vice versa.

### 2.4. Outcomes

Except where otherwise stated, outcomes were common to both the HES and EGPA studies and are reported at the Country-level. In addition, data from the HES and EGPA studies were pooled post hoc to assess overall and country-level outcomes. Analyses of physician characteristics included specialty, practice setting, practice size, years in practice, and average number of unique patients seen with HES or EGPA per year. Analyses of patient data from the patient history (at index) included patient demographics and disease characteristics (including age at diagnosis, sex, disease duration, disease subtype [HES], disease phase [EGPA]), and diagnostic assessments.

Analyses of patient data from diagnosis to EOF included comorbidities, treatment patterns (e.g., number of different therapies received, use of OCS, immunosuppressants, cytotoxic agents, biologics, or other therapies), and time from diagnosis to treatment initiation.

Data on clinical outcomes were assessed from index date to EOF for clinical manifestations, flare (HES), relapse (EGPA), overall survival (HES and EGPA), flare-free survival (HES), relapse-free survival (EGPA).

HES flare was defined as the worsening of HES-related clinical symptoms or blood eosinophil count requiring therapy escalation (e.g., additional or a second-line therapy for HES). EGPA relapse was defined as a recurrence or worsening of EGPA symptoms requiring an increasing OCS dose, an increase/change in dose of immunosuppressive therapy, or hospitalization. Other physician-defined indicators of relapse were also captured.

Response (HES) and remission (EGPA) were assessed from diagnosis to EOF. Response among patients with HES included both complete and partial responses. A complete response was defined as physician-reported improvement or resolution of symptoms, with normal blood eosinophil count ≤ 500 cells/µL. A partial response was defined as physician-reported improvement in symptoms and blood eosinophil count, where blood eosinophil count was not yet in the normal range and the patient still required additional therapy. EGPA remission was defined as Birmingham Vasculitis Activity Score of 0 and OCS dose ≤ 4 mg/day, or other physician-defined remissions.

Data on HCRU from index to EOF included hospitalizations, outpatient visits, emergency room (ER) visits, or other visits related to the monitoring of clinical conditions.

A post hoc pooled analysis (summarizing trends for HES and EGPA combined) was performed to assess comorbidities, treatment patterns (OCS and biologic use), clinical manifestations (at the organ class level), and HCRU.

### 2.5. Sample Size and Statistical Analysis

No formal sample size calculation and no hypothesis testing were performed. Results are summarized using descriptive statistics, data are stratified by country in the HES and EGPA cohorts and presented separately per condition, and for the pooled analyses of both conditions, data are presented overall and stratified by country. Flare-free survival in HES, relapse-free survival in EGPA, and overall survival in both conditions, calculated from the date of disease diagnosis to death, were analyzed using the Kaplan–Meier method. Patients were censored at the EOF or at 6 years after the diagnosis date, whichever came first. Restricted mean survival time (RMST) was reported. Data analysis was performed using SAS Enterprise Guide version 7.15 (SAS Institute Inc., Cary, NC, USA).

### 2.6. Ethics

Only anonymized data was collected, and results are from aggregate analyses that omitted patient identification; therefore, informed consent and ethics committee approval were not required. The studies were submitted to the Western Copernicus Group Institutional Review Board and were granted exemption status with a waiver of authorization issued (EGPA waiver #1-1382529-1; HES waiver #1-1383121-1). Data collection complied with general data protection regulation (GDPR) requirements.

## 3. Results

### 3.1. Physician Characteristics

In the HES study, 22 to 26 physicians participated per country (Table S1). Hematology was the most common specialty among physicians in France (13/25, 52.0%), Germany (11/23, 47.8%), and the UK (17/22, 77.3%), while rheumatology and internal medicine were most common in Italy (7/26, 26.9%) and Spain (7/25, 28.0%), respectively. Italy was the only country with completing physicians from all six targeted specialties: allergy, immunology, rheumatology, pulmonology, hematology, and internal medicine.

In the EGPA study, 38 to 45 physicians from each country participated. Rheumatology was the most common specialty among physicians in Germany (20/39, 51.3%), Spain (15/38, 39.5%), Italy (16/41, 39%), and the UK (29/45, 64.4%), while pulmonology was the most common in France (28/41, 68.3%).

Across all countries, for both HES and EGPA, physicians completed an average of two patient chart reviews. The characteristics of the participating physicians from each country are summarized in Table S1.

### 3.2. Patient Demographics, Clinical Characteristics, and Comorbidities

Detailed patient demographics and clinical characteristics by country are shown in Table 1. For the HES study, data were collected for 280 patients (61 from France, 53 from Germany, 52 each from Italy and Spain, and 62 from the UK). The proportions of male patients in France (46/61, 75.4%) and Spain (38/52, 73.1%) were greater compared with Germany (28/53, 52.8%). The mean (standard deviation [SD]) age at diagnosis was highest in France (47.5 [15.3] years) and lowest in the UK (39.7 [18.6] years). Disease duration from diagnosis to EOF varied between a median (interquartile range [IQR]) of 2.3 (1.8, 2.9) years in Germany to 3.0 (2.1, 4.4) years in France, and 3.0 (1.8, 5.1) years in Italy. Idiopathic HES was the most common subtype in all study countries. The median (IQR) absolute blood eosinophil counts varied, with the lowest value in Italy (700.0 [150.0, 2000.0] cells/µL) and the highest value in France (2900.0 [1050.0, 6075.0] cells/µL).

For the EGPA study, data were collected for 407 patients: 80 each from Germany and Italy, 81 each from France and the UK, and 85 from Spain. More than half of the patients with EGPA were male in all countries, except for Spain (42/85, 49.4%). The mean (SD) age at diagnosis was highest in France (45.5 [14.0] years), followed by the UK (45.4 [14.3] years), and lowest in Italy (39.9 [15.4] years). Asthma prevalence among patients with EGPA varied from 56.3% (45/80) in Germany to 81.5% (66/81) in France. Asthma diagnosis typically preceded EGPA diagnosis, with the shortest median (IQR) interval from asthma diagnosis to EGPA diagnosis of 0.1 (0.0, 2.0) years seen in Italy and longest of 4.4 (2.2, 7.3) years in Germany. Across all countries, the eosinophilic EGPA phase was the most common phase (50.0–58.0%), while the vasculitic EGPA phase was the second most common and was most frequently reported in Germany (41.3%).

**Figure 2:**
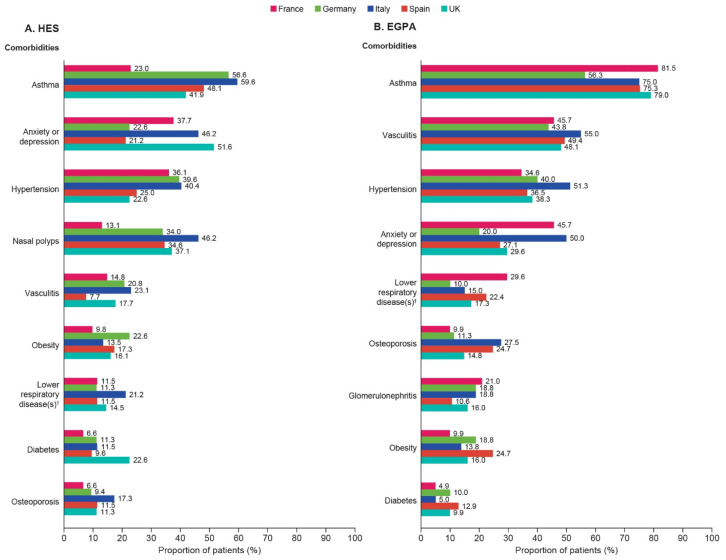
The most common comorbidities among patients with (**A**) HES and (**B**) EGPA. Comorbidity data for the overall HES and overall EGPA populations have been previously published [17,21]. Clinical manifestations, comorbidities, and cancer diagnoses were assessed between HES/EGPA diagnosis and EOF. ^†^ Other than asthma and COPD. COPD, chronic obstructive pulmonary disease; EGPA, eosinophilic granulomatosis with polyangiitis; EOF, end of follow-up; HES, hypereosinophilic syndrome; UK, United Kingdom.

In both HES and EGPA, the order of most common comorbidities varied by country (Figure 2A,B). Anxiety or depression, asthma, hypertension, and nasal polyps were generally more prevalent than other comorbidities in patients with HES in all countries (Figure 2A), whereas among patients with EGPA, asthma, hypertension, anxiety or depression, and vasculitis were generally more prevalent than other comorbidities (Figure 2B). With the exception of Germany, where a similar proportion of patients had comorbid asthma in HES and EGPA, the prevalence of asthma was consistently higher in patients with EGPA than HES.

### 3.3. Diagnostic Assessments and Monitoring

The most common diagnostic test among patients with HES performed across all countries was blood eosinophil count, with the lowest rate of 88.5% in Italy and the highest rate of 96.2% in Spain (Figure S1A).

The most common diagnostic assessments among patients with EGPA conducted across countries were blood tests to detect eosinophilia or autoimmunity. Physicians in Italy were less likely to perform blood tests to detect autoimmunity than other countries (Figure S1B).

### 3.4. Treatment Patterns

A median of 2.0 distinct HES therapies were used per patient across all study countries (Table 2). Most common was OCS, used by the majority of patients in all countries (ranging from 80.3% in France to 100% in Germany). The mean (SD) maximum daily dose for maintenance OCS was highest in Spain (42.4 [19.6] mg/day) and lowest in Italy (24.8 [16.8] mg/day). However, patients from Italy had the longest median (IQR) duration of maintenance OCS, at 23.1 (8.6, 59.0) months. In addition, most patients received immunosuppressants or cytotoxic agents (Table 2), with the highest proportion in France (44/61, 72.1%) and lowest in the UK (34/62, 54.8%). Use of biologics was lowest in Germany (17/53, 32.1%), where the most commonly used biologic was benralizumab, and was highest in the UK (31/62, 50.0%), where alemtuzumab was most common (Table 2). At the EOF, the proportion of patients with HES receiving ongoing OCS therapy decreased substantially in all countries but remained lowest in France (17/61, 27.9%) and highest in Germany (29/53, 54.7%; Table S2).

Patients with EGPA received more distinct therapies than those with HES. The median (IQR) number of distinct EGPA therapies used per patient was similar across countries, 4.0 (3.0, 5.0) in Spain, UK, and Italy, 4.0 (2.0, 5.0) in France, and 3.0 (2.0, 4.0) in Germany. Among patients with EGPA, OCS use was nearly universal, ranging from 97.5% in France to 100.0% in Germany and the UK (Table 2). Mean (SD) maximum daily maintenance OCS dose was highest at 35.8 (18.9) mg in France compared with 19.9 (16.2) mg in Italy. Conversely, the use of immunosuppressants/other therapies ranged from 45.7% in France to 76.5% in the UK, and biologics use ranged from 21.3% in Germany to 68.8% in Italy. In France, biologics use was more common than immunosuppressant use (43/81, 53.1% vs. 37/81, 45.7%); rituximab and mepolizumab were the most used biologics (Table 2). At EOF, more than half of patients with EGPA were still receiving ongoing OCS treatment in all countries, with the lowest rate of 56.8% in the UK and the highest rate of 66.7% in France, and immunosuppressant use remained common (ranging from 25/81, 30.9% in France to 47/81, 58.0% in the UK; Table S3).

### 3.5. Clinical Manifestations

**Figure 3:**
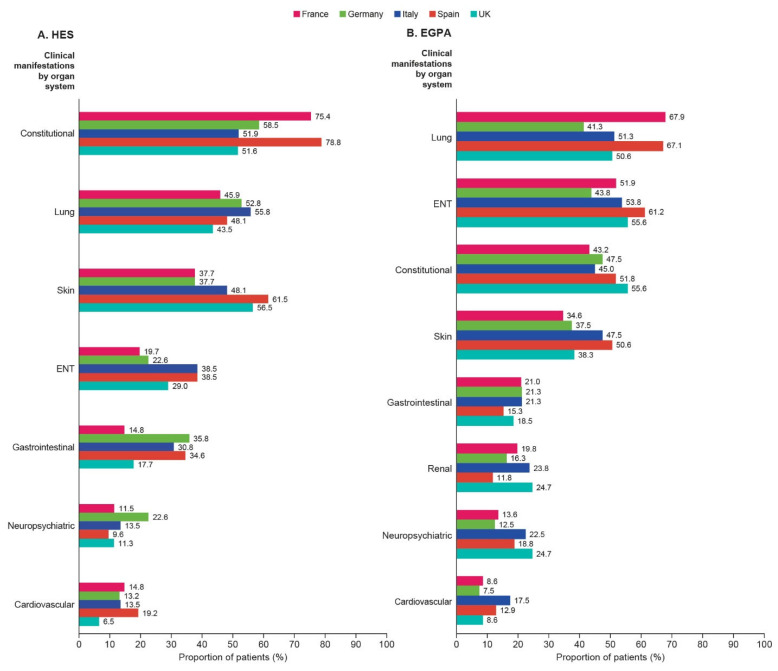
Clinical manifestations by organ system in patients with (**A**) HES and (**B**) EGPA. Clinical manifestation data for the overall HES and overall EGPA populations have been previously published [17,21]. ENT, ear, nose, and throat; EGPA, eosinophilic granulomatosis with polyangiitis; HES, hypereosinophilic syndrome; UK, United Kingdom.

The median (IQR) number of clinical manifestations in patients with HES varied from 2.0 (1.0, 4.0) in the UK to 4.0 (2.0, 6.5) in Spain. Across all countries, over 20% of patients experienced ≥5 distinct clinical manifestations (lowest in France at 13/61, 21.3%, highest in Spain at 21/52, 40.4%). Constitutional (such as fatigue, pain and chills/sweats), lung, and skin manifestations were most prevalent among all the manifestations by organ systems in patients with HES across all countries, although there was considerable country-by-country variation (Figure 3A).

The median (IQR) number of clinical manifestations in patients with EGPA ranged from 2.0 (1.0, 5.0) in Germany to 4.0 (1.0, 6.0) in the UK. Despite some country-by-country variation, the top four most commonly affected organ systems were the lungs, ear, nose, and throat (ENT), constitutional, and skin across all countries (Figure 3B). Individual symptoms and symptom severity in patients with HES and EGPA are shown in Tables S4 and S5.

### 3.6. Clinical Outcomes

The clinical outcomes in patients with HES and EGPA are summarized in Table 3. The proportion of patients with HES experiencing flares (between index date and EOF) was greatest in Italy (17/52, 32.7%) and smallest in France (10/61, 16.4%). The mean (SD) flare frequency per year ranged from 0.3 (0.2) in Spain to 0.7 (0.6) in France. The cumulative median (IQR) duration of flare(s) varied from 1.2 (1.0, 2.9) months in Italy to 4.3 (2.0, 6.9) months in Spain.

Among patients with HES, the rate of clinical responses (between diagnosis and EOF) ranged from 59.7% to 90.6%, with the highest rate in Germany (52.8% complete response; 37.7% partial response) and the lowest rate in the UK (33.9% complete response; 29.0% partial response). The mean 6-year flare-free survival was shortest in Spain (4.64 years) and longest in France (5.14 years) (Figure S2). Overall survival is shown in Figure S3; in most countries there was one death within 6 years of follow-up: one death in France, Germany, Italy, and the UK, and two deaths in Spain.

For EGPA, the proportion of patients who experienced remission was highest in Germany (57/80, 71.3%) and lowest in France (41/80, 50.6%). The proportion of patients with EGPA who experienced relapse was lowest in Germany (7/80, 8.8%) and highest in Spain (25/85, 29.4%). Relapse-free survival ranged from 4.74 years (Spain) to 5.45 years (Germany); Figure S4. Six-year overall survival rate was high across all countries, with deaths ranging from none to five during RMST (Figure S5).

### 3.7. HES and EGPA-Related HCRU

The proportions of patients with HES requiring outpatient, hospitalization, or ER visits across the five countries are shown in Table S6. The proportion requiring outpatient visits was highest in France (57/61, 93.4%) and lowest in Italy (41/52, 78.8%); the proportion requiring hospitalization was highest in the UK (27/62, 43.5%) and lowest in Spain (6/52, 11.5%); and the proportion requiring ER visits was highest in the UK (23/62, 37.1%) and lowest in Italy (7/52, 13.5%).

For EGPA, the proportion of patients with outpatient visits was highest in Italy (75/80, 93.8%); the rates of hospitalization (39/85, 45.9%) and ER visits (52/85, 61.2%) were highest in Spain. The rates of outpatient visits, hospitalization, and ER visits (51/80, 63.8%, 19/80, 23.8%, and 9/80, 11.3%, respectively) were lowest in Germany (Table S7).

### 3.8. Post Hoc Pooled Analysis of HES and EGPA

In the post hoc pooled analysis of patients with HES or EGPA, OCS use was highly prevalent across all countries (94.9% overall, ranging from 90.1% in France to 100% in Germany). Immunosuppressive treatments and biologics were used by 55.9% and 42.9% of overall patients, respectively. Immunosuppressive treatment was more common than biologics use in all countries except for France (Table S8).

The most common comorbidity was asthma (425/687, 61.9% of overall patients; 56.3, 56.4%, and 68.9% of patients in France, Germany, and Italy, respectively), followed by hypertension (254/687, 37.0%), vasculitis (244/687, 35.5%), anxiety and depression (242/687, 35.2%), and lower respiratory diseases (116/687, 16.9%) (Table S9).

In the pooled analysis across all countries, constitutional manifestations occurred in 43.2–55.6% of patients, manifestations of the lung in 37.8–54.0%, ENT manifestations in 31.7–48.2%, and skin manifestations in 26.1–39.4% (Table S10). The proportion of patients with clinical manifestations of the lung was lowest in the UK (54/143, 37.8%) and highest in Spain (74/137, 54.0%). Presentation of ENT was also highest in Spain (66/137, 48.2%) and was lowest in France (45/142, 31.7%; Table S10).

For both HES and EGPA across the five countries, 70.7–91.2% of patients had disease-related outpatient visits; 15.0–47.4% had ER visits; and 25.6–37.9% had hospitalizations between the index date and EOF. HCRU was consistently lowest in Germany (Table S11). Patients with HES and EGPA may experience tests related to complications and adverse effects associated with immunosuppressive medications, including imaging tests (253/687, 36.8%), bone mineral density testing (233/687, 33.9%), and cataract removal (22/687, 3.2%) (Table S11).

## 4. Discussion

This study provides real-world insight into two rare eosinophilic diseases, utilizing data from five European countries, allowing a better understanding of treating physician and patient characteristics, diagnosis and treatment, clinical manifestations, clinical outcomes, and HCRU. Such data have been scarce from previous studies, particularly in Europe. Variations were observed in the primary physician specialties treating patients with HES and EGPA across the five countries. Although only primary physician specialty was reported, patients with HES and EGPA often require multidisciplinary treatment, and data on the broader care team were not captured [2]. Patient profiles across the countries showed broad similarities, yet distinctions in sex and age at diagnosis emerged. Extensive OCS use was observed across all patients with HES and EGPA; however, biologics use varied by country. As expected, manifestations were heterogeneous, but fatigue and lung manifestations were frequently reported among all patients. Clinical outcomes varied by country, but in all countries a notable proportion of patients did not achieve a complete response (HES) or remission (EGPA); flares (HES) and relapses (EGPA) were common and HCRU was high.

Consistently, in the pooled analysis of patients with HES or EGPA, over 90% of patients across countries were treated with OCS, with a long median duration of treatment from 17.6 to 27.3 months and high maximum daily doses, indicating a reliance on OCS and exposing patients to a significant risk of adverse effects [14,24,25]. Long-term OCS use has been associated with a range of adverse effects including diabetes mellitus, secondary hypertension and osteoporosis, adrenal complications, and mood and sleep disorders, the risks of which increase with dosage and which can further impact HCRU [14,15,16,24,26]. Despite the extensive use of OCS and other immunosuppressive or cytotoxic therapies in this study, rates of relapse and flares were high and a substantial proportion of patients did not achieve response or remission, indicating suboptimal disease control. These results are consistent with previous findings in patients with EGPA that short term remission is frequently followed by relapse, often when OCS tapering occurs [27]. These findings highlight the need for early intervention with well-tolerated and effective treatment options, especially for patients with refractory and/or relapsing disease [28,29].

Over the last several years, the availability of targeted therapies including eosinophil-targeting biologics has started to change the treatment landscape for HES and EGPA [2,3,13,30,31]. In this group, mepolizumab is a first-in-class humanized monoclonal antibody that specifically targets interleukin-5 (IL-5). Mepolizumab is approved for the treatment of severe asthma with an eosinophilic phenotype, EGPA, HES and CRSwNP in multiple regions worldwide, having received EMA approval for HES and EGPA in 2021 [32]. More recently, benralizumab, a humanized monoclonal antibody targeting the IL-5 receptor, has been approved for asthma and (since 2024) EGPA [33]. The most commonly used biologics in the pooled analysis were mepolizumab (17%), benralizumab (9%) and rituximab (16%), an anti-CD20 B-cell targeted therapy which is recommended as an off-label treatment for EGPA [2,11]. Other biologics targeting the IL-5 pathway are also under investigation for HES and EGPA [30,31]. Consistent with a previous retrospective study of off-label biologic use in HES, we found that a variety of other biologic therapies had been used (off-label) in the treatment of both HES and EGPA [34].

Although the relationship between treatment intervention and disease control was not investigated in the current analysis, past research has demonstrated the benefits of biologics in this area. Phase III randomized controlled trials of mepolizumab for HES and EGPA have demonstrated efficacy versus placebo in addition to standard of care. Patients with HES had a 66% reduction in risk of flare and a reduction in symptom severity with mepolizumab versus placebo [35,36]. Patients with EGPA receiving mepolizumab versus placebo spent significantly more time in remission, had a 50% reduction in relapse rate and reduced OCS use [37]. Long-term use of mepolizumab was also well tolerated and resulted in sustained treatment benefits [38,39]. Similar remission and relapse results were seen for benralizumab in a phase III non-inferiority trial versus mepolizumab [40]. Real-world studies further support the clinical benefits of biologics such as mepolizumab. Mepolizumab was associated with improved disease control (reduced relapse rates, improved symptoms or reduced EGPA-related hospitalizations) and reduced corticosteroid use post- versus pre-mepolizumab initiation in patients with EGPA in both Japan and the US [38,41], and with remission maintenance, improved flare control and reduced treatment-related morbidity in patients with HES [42,43]. Despite the advancement in HES and EGPA treatment options over the last several years, only 42% of patients received biologics and there was substantial variation between countries. This combined with the evidence of suboptimal disease control despite high levels of OCS use seen in this study suggests an opportunity for broader and earlier intervention with precision targeted therapies to enhance treatment outcomes.

Although treatment decisions were not directly assessed in this study, differences in treatment use could be influenced by a number of factors. EULAR treatment recommendations are dependent on EGPA severity and manifestations but also emphasize the importance of considering comorbidities, patient history, toxicity and tolerability, patient preference and local availability and cost of treatments [11]. For patients with HES, identification of disease subtype and any underlying genetic causes is a key determinant of treatment approach [13]. There are many areas of future research to be addressed in order to improve the evidence base for treatment decisions and thereby improve care for patients with EGPA and HES, including understanding the impact of timing of interventions, different therapy combinations and how patients’ characteristics, disease phenotype, and biomarkers affects response to therapy [11,29,31]. The impact of baseline blood eosinophil count and ANCA status on treatment outcomes with eosinophil targeted therapies has been investigated; for EGPA more pronounced treatment benefits with mepolizumab versus placebo were demonstrated among patients with baseline blood eosinophil count ≥150 cells/µL, but both mepolizumab and benralizumab show clinical benefits irrespective of ANCA status [37,44,45]. For patients with HES, although there is a trend for patients with blood eosinophil count ≥2500 cells/µL to experience the greatest improvements with mepolizumab, no significant interaction was found between baseline blood eosinophil count and treatment effect [46].

The most frequent HES and EGPA clinical manifestations across all five countries included fatigue and manifestations of the lung, ENT, and skin. The nature of the clinical manifestations were similar to those reported in a US cohort study for EGPA [9] and a retrospective chart review for HES [10]. In the pooled analysis, pain was most commonly reported in Spain and cardiovascular manifestations were twice as common in Spain and Italy than in the UK. The proportion of patients with HES who experienced clinical responses and the proportion of patients with EGPA who experienced remission varied by country. A number of factors may explain these variations; environmental and genetic risk factors for the development of EGPA have been previously identified, and the latter can drive the type of manifestations seen [47,48,49,50,51]; however, genetic risk factors have not been shown to vary at the population level. Additionally, differences in disease awareness, clinical practice by healthcare professionals and healthcare/treatment access across countries could also contribute to variations in reported manifestations and clinical outcomes [22,52,53]. In the pooled analysis, the biggest inter-country variations in HCRU were seen in emergency visits, which were as low as 15% in Germany but as high as 47% in Spain, which may reflect differences in disease control or healthcare system structure. The considerable HCRU burden illustrated in this study highlights the need for more optimal patient management to improve clinical outcomes and reduce the burden on patients and the healthcare system. In addition to the aforementioned need for further research on treatment stratification and earlier intervention with targeted therapies, improved disease management may include earlier and accurate diagnosis, which is often delayed for patients with HES and EGPA, and clearer care pathways aligned with published recommendations for a multi-disciplinary approach [1,2,29].

This analysis leveraged two studies across five European countries to assess a significant number of patients with two rare diseases—HES and EGPA. To date, this is the largest real-world evidence analysis of these conditions across countries with different healthcare systems. This analysis adds to the existing literature by expanding on the known disease burden of HES and EGPA, including the high frequency of medical visits and hospitalizations, as highlighted in prior research [17,18,19]. The analyses reported real-world longitudinal data from five European countries, which offers insights into the heterogeneity of the populations studied and results that may be generalizable to patients with HES and EGPA in similar healthcare systems. Differences in country-specific healthcare policies, including reimbursement and access to biologics, may also contribute to the observed variation in treatment patterns.

A key limitation of this study was that data collection was limited to the maximum daily dose and duration of OCS use, and it was not possible to calculate average daily dose over a standardized time period. These measures could be influenced by the availability of alternative treatments and patient disease severity. A large range between countries in maximum OCS dose was observed, which also may not be reflected in mean daily OCS use over a standardized period. In addition, information regarding treatment start and stop dates, the timing of flares, relapses and responses was not available, and so temporal trends including changes in therapy patterns over time or the sequence of treatments and association with outcomes cannot be determined. Other limitations of these studies have been previously discussed and addressed, including their retrospective nature, potential for reporting bias, selection bias, and non-random missing data, variability of information recorded on medical charts between countries, and inability to audit medical records and verify accuracy of data entries because of physician anonymity [17,21]. As diagnoses of EGPA and HES were based on physician judgment without a requirement for standardized diagnostic criteria, the potential for misclassification bias should also be considered. Additionally, mild flares that did not lead to therapy adjustments may have been underreported, potentially underestimating flare and relapse frequency.

## 5. Conclusions

This analysis identified a substantial burden of disease, extensive OCS use, and notable rates of hospitalizations and ER visits for patients with HES and EGPA across five European countries. Results highlight the need for further improvements in disease management and more uniform approaches across Europe for treating these patients with rare diseases.

## Figures and Tables

**Table 1:** Baseline demographics and clinical characteristics * for patients with HES and EGPA.

Patients with HES (N = 280)	France n = 61	Germany n = 53	Italy n = 52	Spain n = 52	UK n = 62
**Length of follow-up, years,** mean (SD)	3.1 (1.4)	2.3 (1.1)	2.8 (1.5)	3.0 (1.4)	2.5 (1.3)
**Male,** n (%)	46 (75.4)	28 (52.8)	30 (57.7)	38 (73.1)	40 (64.5)
**Age on index date, years,** mean (SD)	48.5 (14.5)	42.3 (17.7)	42.3 (15.1)	43.6 (12.1)	41.3 (18.1)
**Age at HES diagnosis, years,** mean (SD)	47.5 (15.3)	42.0 (17.9)	40.2 (14.8)	42.3 (12.6)	39.7 (18.6)
**Disease duration, years, from** **diagnosis date to EOF**					
Mean (SD)	4.1 (4.4)	2.6 (1.6)	4.9 (5.9)	4.3 (4.2)	4.1 (4.9)
Median (IQR)	3.0 (2.1, 4.4)	2.3 (1.8, 2.9)	3.0 (1.8, 5.1)	2.9 (1.7, 4.5)	2.4 (1.7, 4.1)
**HES diagnosis before study (≤2014),** n (%)	7 (11.5)	1 (1.9)	9 (17.3)	7 (13.5)	8 (12.9)
**HES diagnosis during the study (2015–2019),** n (%)	54 (88.5)	52 (98.1)	43 (82.7)	45 (86.5)	54 (87.1)
**Disease subtype,** ^†^ n (%)					
Idiopathic	33 (54.1)	30 (56.6)	32 (61.5)	27 (51.9)	33 (53.2)
Myeloid variant	16 (26.2)	10 (18.9)	9 (17.3)	18 (34.6)	13 (21.0)
Lymphocytic variant	11 (18.0)	8 (15.1)	4 (7.7)	5 (9.6)	14 (22.6)
Other ^†,‡^	0 (0.0)	0 (0.0)	1 (1.9)	0 (0.0)	1 (1.6)
Unknown	1 (1.6)	5 (9.4)	6 (11.5)	2 (3.8)	1 (1.6)
**Blood eosinophil count data** **available, **^§^ n (%)	56 (91.8)	44 (83.0)	45 (86.5)	45 (86.5)	51 (82.3)
Blood eosinophil count, ^§^ cells/µL					
Mean (SD)	3641.3 (2837.0)	2242.6 (1730.1)	1412.1 (1573.3)	2920.6 (2233.7)	2303.4 (2694.1)
Median (IQR)	2900.0 (1050.0, 6075.0)	1650.0 (1000.0, 3100.0)	700.0 (150.0, 2000.0)	2479.0 (1600.0, 3200.0)	1600.0 (89.0, 3500.0)
**Patients with EGPA (N = 407)**	**France** **n = 81**	**Germany** **n = 80**	**Italy** **n = 80**	**Spain** **n = 85**	**UK** **n = 81**
**Length of follow-up, years,** mean (SD)	2.4 (1.3)	2.6 (1.3)	3.2 (1.6)	2.9 (1.5)	2.6 (1.4)
**Male,** n (%)	51 (63.0)	44 (55.0)	47 (58.8)	42 (49.4)	47 (58.0)
**Age on index date, years,** mean (SD)	45.8 (13.9)	42.6 (15.8)	41.4 (16.1)	44.2 (14.8)	46.1 (14.0)
**Age at EGPA diagnosis, years,** mean (SD)	45.5 (14.0)	42.3 (15.6)	39.9 (15.4)	42.8 (14.8)	45.4 (14.3)
**Disease duration, years,** from diagnosis date to EOF					
Mean (SD)	2.7 (1.7)	3.0 (2.1)	4.6 (3.9)	4.2 (4.2)	3.3 (3.1)
Median (IQR)	2.1 (1.8, 2.9)	2.2 (1.7, 3.1)	3.7 (2.0, 5.9)	2.8 (1.9, 4.4)	2.3 (1.7, 3.5)
**EGPA diagnosis before study (≤2014),** n (%)	4 (4.9)	4 (5.0)	19 (23.8)	12 (14.1)	9 (11.1)
**EGPA diagnosis during the study** **(2015–2019),** n (%)	77 (95.1)	76 (95.0)	61 (76.3)	73 (85.9)	72 (88.9)
**Proportion of patients with asthma**, n (%)	66 (81.5)	45 (56.3)	60 (75.0)	64 (75.3)	64 (79.0)
Proportion of patients with an asthma diagnosis date prior to EGPA diagnosis, ^¶^ n (%)	39 (78.0)	24 (100.0)	36 (80.0)	33 (64.7)	28 (80.0)
Time from asthma diagnosis to EGPA diagnosis, years					
Mean (SD)	2.3 (3.2)	8.2 (11.3)	1.9 (3.9)	5.6 (6.2)	5.5 (5.6)
Median (IQR)	1.2 (0.3, 3.2)	4.4 (2.2, 7.3)	0.1 (0.0, 2.0)	2.9 (0.5, 8.1)	3.6 (0.9, 9.2)
**Disease phase,** ^†^ n (%)					
Eosinophilic	47 (58.0)	40 (50.0)	44 (55.0)	48 (56.5)	41 (50.6)
Vasculitic	21 (25.9)	33 (41.3)	28 (35.0)	20 (23.5)	23 (28.4)
Prodromal	9 (11.1)	4 (5.0)	4 (5.0)	9 (10.6)	10 (12.3)
Unknown	4 (4.9)	3 (3.8)	4 (5.0)	8 (9.4)	7 (8.6)
**Blood eosinophil count data** **available, ^§^** n (%)	77 (95.1)	73 (91.3)	66 (82.5)	75 (88.2)	73 (90.1)
Blood eosinophil count, ^§^ cells/µL					
Mean (SD)	2125.6 (2035.0)	3098.1 (2266.2)	2345.6 (2123.2)	2345.7 (2399.2)	1948.1 (2542.1)
Median (IQR)	1500.0 (875.0, 2800.0)	2800.0 (1200.0, 4500.0)	1800.0 (900.0, 3000.0)	1400.0 (600.0, 4000.0)	800.0 (45.0, 3200.0)

* Baseline demographics and clinical characteristics data for the overall HES and overall EGPA population have been previously published [17,21]. ^†^ Based on physician assessment, no criteria were specified. ^‡^ Other disease subtype reported as chronic eosinophilic leukemia in both cases. ^§^ Physicians reported the most recent documented lab test values for the patient between the diagnosis date and index date. ^¶^ Calculated out of the number of patients who had a reported diagnosis date for asthma: France (n = 50), Germany (n = 24), Italy (n = 45), Spain (n = 51), and the UK (n = 35); 94 patients who had asthma between EGPA diagnosis and EOF did not have a reported asthma diagnosis date. In total, 45 patients had an asthma diagnosis after EGPA diagnosis and 94 patients with asthma did not have a reported asthma diagnosis date. EGPA, eosinophilic granulomatosis with polyangiitis; EOF, end of follow-up; HES, hypereosinophilic syndrome; IQR, interquartile range; SD, standard deviation; UK, United Kingdom.

**Table 2:** Treatment use * in patients with HES and EGPA.

Patients with HES ^†^ (N = 280)	France n = 61	Germany n = 53	Italy n = 52	Spain n = 52	UK n = 62
**Number of distinct HES therapies used,** ^‡^ median (IQR)	2.0 (2.0, 4.0)	2.0 (2.0, 3.0)	2.0 (1.0, 3.0)	2.0 (2.0, 3.0)	2.0 (2.0, 3.0)
**Number of distinct HES therapies used, ^‡^** categorical, n (%)					
0	2 (3.3)	0 (0.0)	1 (1.9)	0 (0.0)	1 (1.6)
1	8 (13.1)	13 (24.5)	15 (28.8)	12 (23.1)	12 (19.4)
2	22 (36.1)	20 (37.7)	14 (26.9)	22 (42.3)	29 (46.8)
3	13 (21.3)	11 (20.8)	13 (25.0)	14 (26.9)	14 (22.6)
≥4	16 (26.2)	9 (17.0)	9 (17.3)	4 (7.7)	6 (9.7)
**HES therapies by treatment category** ^§^					
** OCS, n (%)**	**49 (80.3)**	**53 (100)**	**45 (86.5)**	**50 (96.2)**	**53 (85.5)**
Maximum daily dose across all OCS, mg, ^¶^ mean (SD)	31.6 (20.2)	28.0 (18.1)	24.8 (16.8)	42.4 (19.6)	32.0 (17.7)
Total duration across all oral corticosteroids, months					
Mean (SD)	17.5 (19.0)	22.3 (18.7)	43.5 (54.3)	22.0 (18.3)	17.9 (12.0)
Median (IQR)	8.5 (5.7, 24.1)	21.3 (10.2, 28.7)	23.1 (8.6, 59.0)	18.4 (5.8, 30.8)	15.9 (10.4, 23.6)
Prednisone or prednisolone, n (%)	39 (63.9)	46 (86.8)	30 (57.7)	43 (82.7)	42 (67.7)
Methylprednisolone, n (%)	4 (6.6)	4 (7.5)	18 (34.6)	8 (15.4)	12 (19.4)
Cortisone, n (%)	7 (11.5)	3 (5.7)	1 (1.9)	0 (0)	3 (4.8)
**Immunosuppressants or cytotoxic agents, used by ≥5% of patients in any country,** ** n (%)	**44 (72.1)**	**33 (62.3)**	**34 (65.4)**	**33 (63.5)**	**34 (54.8)**
Azathioprine	8 (13.1)	6 (11.3)	13 (25)	5 (9.6)	8 (12.9)
Cyclophosphamide	3 (4.9)	6 (11.3)	3 (5.8)	2 (3.8)	3 (4.8)
Cyclosporine	2 (3.3)	2 (3.8)	4 (7.7)	2 (3.8)	4 (6.5)
Hydroxyurea	6 (9.8)	6 (11.3)	4 (7.7)	4 (7.7)	6 (9.7)
Imatinib mesylate	16 (26.2)	12 (22.6)	10 (19.2)	11 (21.2)	8 (12.9)
Interferon-alpha	3 (4.9)	8 (15.1)	1 (1.9)	1 (1.9)	2 (3.2)
Methotrexate	7 (11.5)	5 (9.4)	6 (11.5)	10 (19.2)	1 (1.6)
Tofacitinib	2 (3.3)	1 (1.9)	3 (5.8)	0 (0.0)	1 (1.6)
Mycophenolate	0 (0.0)	0 (0.0)	0 (0.0)	3 (5.8)	2 (3.2)
**Biologics**, n (%)	**30 (49.2)**	**17 (32.1)**	**25 (48.1)**	**20 (38.5)**	**31 (50.0)**
Mepolizumab	9 (14.8)	4 (7.5)	16 (30.8)	9 (17.3)	5 (8.1)
Alemtuzumab	1 (1.6)	4 (7.5)	2 (3.8)	2 (3.8)	13 (21)
Benralizumab	9 (14.8)	9 (17)	6 (11.5)	3 (5.8)	7 (11.3)
Dupilumab	5 (8.2)	5 (9.4)	6 (11.5)	0 (0)	6 (9.7)
Omalizumab	1 (1.6)	2 (3.8)	5 (9.6)	5 (9.6)	0 (0)
Reslizumab	7 (11.5)	1 (1.9)	0 (0)	2 (3.8)	3 (4.8)
Rituximab	11 (18)	5 (9.4)	4 (7.7)	9 (17.3)	5 (8.1)
**Patients with EGPA** ^††^ **(N = 407)**	**France** **n = 81**	**Germany** **n = 80**	**Italy** **n = 80**	**Spain** **n = 85**	**UK** **n = 81**
**Number of distinct EGPA therapies used,** ^‡^ median (IQR)	4.0 (2.0, 5.0)	3.0 (2.0, 4.0)	4.0 (3.0, 5.0)	4.0 (3.0, 5.0)	4.0 (3.0, 5.0)
**Number of distinct EGPA therapies used, categorical,** ^‡^ n (%)					
1–2	26 (32.1)	33 (41.3)	9 (11.3)	14 (16.5)	20 (24.7)
3–4	31 (38.3)	30 (37.5)	36 (45.0)	34 (40.0)	35 (43.2)
5–7	19 (23.5)	16 (20.0)	31 (38.8)	36 (42.4)	24 (29.6)
≥8	5 (6.2)	1 (1.3)	4 (5.0)	1 (1.2)	2 (2.5)
**Time from diagnosis to initiation of EGPA therapy,** years					
Mean (SD)	0.3 (1.2)	0.2 (0.6)	0.7 (1.7)	0.9 (2.7)	0.0 (0.1)
Median (IQR)	0.0 (0.0, 0.0)	0.0 (0.0, 0.1)	0.0 (0.0, 0.2)	0.0 (0.0, 0.9)	0.0 (0.0, 0.0)
**Time from diagnosis to initiation of biologics, years,** median (IQR)	1.0 (0.1, 1.5)	0.7 (0.1, 1.6)	2.8 (1.2, 4.6)	1.9 (1.2, 3.0)	0.5 (0.1, 1.8)
**EGPA therapies by treatment category** ^‡‡^					
** OCS, n (%)**	**79 (97.5)**	**80 (100.0)**	**79 (98.8)**	**83 (97.6)**	**81 (100.0)**
Maximum daily OCS dose (mg) ^¶^	35.8 (18.9)	28.8 (19.9)	19.9 (16.2)	34.0 (19.8)	31.6 (20.4)
Duration of OCS use, months					
Mean (SD)	22.3 (15.0)	23.1 (16.8)	38.3 (38.1)	31.6 (39.3)	28.3 (26.3)
Median (IQR)	19.1 (12.3, 27.0)	21.4 (8.9, 29.9)	27.9 (19.2, 47.0)	19.2 (8.2, 38.2)	22.5 (12.7, 39.7)
Prednisone or prednisolone, n (%)	72 (88.9)	75 (93.8)	56 (70.0)	71 (83.5)	75 (92.6)
Methylprednisolone, n (%)	26 (32.1)	7 (8.8)	22 (27.5)	13 (15.3)	30 (37.0)
Cortisone, n (%)	2 (2.5)	1 (1.3)	7 (8.8)	4 (4.7)	5 (6.2)
**Immunosuppressive agents and other therapies, used by ≥5% of patients in any country,** ^§§^ n (%)	**37 (45.7)**	**54 (67.5)**	**56 (70.0)**	**51 (60.0)**	**62 (76.5)**
Azathioprine	14 (17.3)	29 (36.3)	22 (27.5)	16 (18.8)	29 (35.8)
Cyclophosphamide	6 (7.4)	23 (28.8)	21 (26.3)	13 (15.3)	15 (18.5)
Cyclosporine	3 (3.7)	2 (2.5)	2 (2.5)	5 (5.9)	3 (3.7)
Immunoglobulin (intravenous)	5 (6.2)	1 (1.3)	1 (1.3)	2 (2.4)	0 (0.0)
Methotrexate	13 (16.0)	12 (15.0)	17 (21.3)	19 (22.4)	16 (19.8)
Mycophenolate	1 (1.2)	1 (1.3)	3 (3.8)	8 (9.4)	19 (23.5)
**Biologics, used by ≥5% of patients in any country,** ^¶¶^ n (%)	**43 (53.1)**	**17 (21.3)**	**55 (68.8)**	**43 (50.6)**	**27 (33.3)**
Mepolizumab	18 (22.2)	5 (6.3)	31 (38.8)	12 (14.1)	8 (9.9)
Benralizumab	3 (3.7)	3 (3.8)	7 (8.8)	9 (10.6)	4 (4.9)
Omalizumab	1 (1.2)	4 (5.0)	4 (5.0)	6 (7.1)	3 (3.7)
Reslizumab	4 (4.9)	0 (0.0)	3 (3.8)	8 (9.4)	1 (1.2)
Rituximab	26 (32.1)	8 (10.0)	11 (13.8)	15 (17.6)	14 (17.3)

* Treatment use data for the overall HES and overall EGPA population have been previously published [17,21]. ^†^ Treatment patterns for HES therapies were assessed between HES diagnosis and the EOF (i.e., last physician encounter or death). ^‡^ Receipt of one or multiple oral corticosteroid drugs was counted as a single therapy. ^§^ Additional details of HES therapies other than OCS, immunosuppressive and biologic therapies are given in Table S2. ^¶^ Maximum daily dose for maintenance therapy values over 60 mg were removed from the summary statistics, as these values likely reflected dosing for burst treatment episodes instead of maintenance therapy. ** Treatments used by <5% of patients with HES each country were chlorambucil, dexpramipexole, etoposide, peg-interferon, ruxolitinib, vincristine, and leflunomide. ^††^ Treatment patterns for EGPA therapies were assessed between EGPA diagnosis and EOF (i.e., last physician encounter or death). ^‡‡^ Additional details of EGPA therapies other than OCS, immunosuppressive and biologic therapies are given in Table S3. ^§§^ Treatments used by <5% of patients with EGPA in each country were hydroxyurea, interferon-alpha, leflunomide, and plasma exchange. ^¶¶^ Treatments used by <5% of patients within each country were dupilumab. EGPA, eosinophilic granulomatosis with polyangiitis; EOF, end of follow-up; HES, hypereosinophilic syndrome; IQR, interquartile range; OCS, oral corticosteroid; SD, standard deviation; UK, United Kingdom.

**Table 3:** Clinical outcomes * for patients with HES and EGPA.

Patients with HES (N = 280)	France n = 61	Germany n = 53	Italy n = 52	Spain n = 52	UK n = 62
**Flares (from index date to EOF)** ^†^					
Patients who experienced a flare, n (%)	10 (16.4)	10 (18.9)	17 (32.7)	14 (26.9)	13 (21.0)
Number of flares per year ^‡^					
Mean (SD)	0.7 (0.6)	0.6 (0.3)	0.5 (0.2)	0.3 (0.2)	0.6 (0.3)
Median (IQR)	0.4 (0.3, 0.7)	0.5 (0.4, 1.0)	0.5 (0.3, 0.6)	0.3 (0.2, 0.4)	0.6 (0.3, 0.7)
Cumulative duration of flare(s), ^‡^ months					
Mean (SD)	4.8 (6.3)	2.4 (1.6)	2.1 (1.8)	4.2 (2.9)	1.6 (1.2)
Median (IQR)	2.4 (1.0, 5.5)	2.2 (1.1, 3.7)	1.2 (1.0, 2.9)	4.3 (2.0, 6.9)	2.0 (0.4, 2.4)
Time to first flare from diagnosis, months					
Mean (SD)	23.2 (15.6)	11.4 (5.1)	44.1 (52.7)	30.6 (22.6)	32.7 (34.7)
Median (IQR)	24.0 (6.6, 36.5)	11.1 (6.3, 15.0)	22.0 (14.7, 56.8)	29.8 (15.2, 35.9)	25.1 (8.6, 41.1)
**Responses (from diagnosis to EOF)** ^§^					
Patients who experienced a response, n (%) ^¶^	43 (70.5)	48 (90.6)	34 (65.4)	38 (73.1)	37 (59.7)
Duration of response(s), cumulative, ^¶^ months					
Mean (SD)	19.3 (15.0)	18.9 (17.6)	14.3 (13.1)	14.2 (12.1)	11.7 (10.9)
Median (IQR)	17.3 (6.0, 28.6)	17.2 (5.5, 25.9)	10.2 (2.0, 23.0)	13.7 (5.5, 18.3)	9.8 (3.6, 15.8)
Time to first response from diagnosis, months					
Mean (SD)	14.0 (14.1)	11.2 (9.9)	33.2 (54.4)	19.3 (23.4)	23.6 (32.4)
Median (IQR)	8.5 (4.9, 20.9)	9.2 (2.4, 19.3)	15.7 (4.9, 36.1)	9.9 (4.6, 22.6)	15.2 (4.1, 26.9)
Patients who experienced a complete response, n (%)	26 (42.6)	28 (52.8)	19 (36.5)	19 (36.5)	21 (33.9)
Patients who experienced a partial response, n (%)	17 (27.9)	20 (37.7)	15 (28.8)	19 (36.5)	18 (29.0)
**Patients with EGPA (N = 407)**	**France** **n = 81**	**Germany** **n = 80**	**Italy** **n = 80**	**Spain** **n = 85**	**UK** **n = 81**
**Remission status (from diagnosis to EOF)**					
Patients who experienced remission, n (%)	41 (50.6)	57 (71.3)	42 (52.5)	57 (67.1)	45 (55.6)
Duration of remission(s), cumulative, months ^¶^					
Mean (SD)	16.4 (16.9)	20.8 (15.6)	12.3 (12.9)	14.4 (16.5)	16.0 (13.4)
Median (IQR)	11.7 (3.9, 20.6)	15.7 (10.0, 27.8)	6.9 (2.3, 16.2)	8.3 (4.0, 20.0)	12.0 (7.4, 22.2)
Time to first remission from diagnosis, months					
Mean (SD)	16.4 (12.7)	13.8 (20.5)	38.0 (45.1)	35.9 (46.2)	19.8 (33.0)
Median (IQR)	14.8 (6.9, 23.8)	8.1 (3.8, 14.0)	27.0 (17.9, 36.8)	19.1 (12.9, 37.0)	9.7 (6.9, 17.0)
Remission criteria used, n/N (%) **					
BVAS = 0	5/26 (19.2)	7/31 (22.6)	4/27 (14.8)	5/30 (16.7)	3/32 (9.4)
OCS dosage use of ≤4.0 mg/day	17/26 (65.4)	23/31 (74.2)	18/27 (66.7)	20/30 (66.7)	21/32 (65.6)
Other ^††^	3/26 (11.5)	1/31 (3.2)	1/27 (3.7)	4/30 (13.3)	8/32 (25.0)
Missing	3/26 (11.5)	4/31 (12.9)	4/27 (14.8)	3/30 (10.0)	5/32 (15.6)
**Relapse status (from index date to EOF) ^‡‡^**					
Patients who experienced relapse, n (%)	13 (16.0)	7 (8.8)	15 (18.8)	25 (29.4)	18 (22.2)
Number of relapse (among patients with a relapse per person per year)					
Mean (SD)	0.6 (0.3)	0.5 (0.4)	0.9 (0.4)	0.9 (0.8)	1.1 (1.9)
Median (IQR)	0.6 (0.3, 0.6)	0.4 (0.3, 0.5)	0.8 (0.6, 1.2)	0.6 (0.3, 0.9)	0.5 (0.3, 1.0)

* Clinical outcome data for the overall HES and overall EGPA population have been previously published [17,21]. ^†^ A flare was defined as the worsening of HES-related clinical symptoms or blood eosinophil count requiring therapy escalation (e.g., increase in the dose of the current therapy or addition of new therapy). ^‡^ Summary statistics for the number of flares per year were reported among the patients who had ≥1 flare. For patients who had ≥1 flare from diagnosis to the EOF, the duration of flare was calculated as the sum of durations of flare for all reported flares. ^§^ Responses included both complete and partial responses. A complete response was defined as having physician-reported improved or resolved symptoms and normal blood eosinophil count (≤500 cells/μL). A partial response was defined as having physician-reported improved symptoms and blood eosinophil count, where blood eosinophil count is not yet in the normal range and the patient still requires additional therapy. Responses were assessed between HES diagnosis date and the EOF (i.e., last physician encounter or death). A small share of patients who had >1 different responses reported had both a complete response and a separate response reported. ^¶^ Summary statistics for the number of responses were reported among the patients who had ≥1 response. For patients who had ≥1 response, the duration of response was calculated as the sum of durations of response for all reported responses. ** Assessed among physicians with ≥1 patients who experienced remission. Physicians could report one or more remission definitions. ^††^ The other definitions of remission included BVAS = 2 (1), normal ANCA (3), normal ESR (2), no clinical sign (9), clinical control (4), clinical improvement (1), prednisone <10 mg/day (1), decrease in corticosteroid dosage (2), and disappearance of the infiltrates (1). Two physicians did not specify remission definition. ^‡‡^ Relapse was defined as a recurrence or worsening of EGPA symptoms requiring an increasing OCS dose, an increase/change in dose of immunosuppressive therapy, or hospitalization. Indication of relapse based on other definitions was also included. ANCA, antineutrophilic cytoplasmic antibody; BVAS, Birmingham vasculitis activity score; EGPA, eosinophilic granulomatosis with polyangiitis; EOF, end of follow-up; ESR, erythrocyte sedimentation rate; HES, hypereosinophilic syndrome; IQR, interquartile range; OCS, oral corticosteroid; SD, standard deviation; UK, United Kingdom.

## Data Availability

Restrictions apply to the availability of these data. Data were obtained from GSK via Analysis Group, Inc., and are not publicly available due to contractual and privacy obligations. Qualified researchers may request access to minimal anonymized datasets by contacting the corresponding author. Any data sharing is subject to approval by the data custodian (GSK) and may require a data use agreement.

## Supplementary Materials

The following supporting information can be downloaded at: https://www.mdpi.com/article/10.3390/jcm14124309/s1, Figure S1: Use of select diagnostic tests and assessments * in A. patients with HES and B. patients with EGPA; Figure S2: Flare-free survival in patients with HES by country (A-E); Figure S3: Overall survival in patients with HES by country (A-E); Figure S4: Relapse-free survival for patients with EGPA by country (A-E); Figure S5: Overall survival in patients with EGPA by country (A-E); Table S1: Characteristics of participating physicians treating patients with HES or EGPA; Table S2: Other treatment use in patients with HES and ongoing HES therapies at EOF; Table S3: Other treatment use in patients with EGPA and ongoing treatments at the EOF; Table S4: Clinical manifestations and symptom severity in patients with HES; Table S5: Clinical manifestations and symptom severity in patients with EGPA; Table S6: HCRU in HES; Table S7: HCRU in EGPA; Table S8: Pooled analysis of treatment use in patients with HES or EGPA overall and by country; Table S9: Pooled comorbidities data among patients with HES and EGPA (overall and by country); Table S10: Pooled clinical manifestations and symptom severity in HES and EGPA, overall and by country; Table S11: Pooled HCRU for HES and EGPA, overall and by country.
