# Supplementary material for: High Disease Burden and Oral Corticosteroid Use in Patients with Hypereosinophilic Syndrome and Eosinophilic Granulomatosis with Polyangiitis: Country-Level Insights into Real-World Management in Europe
Source: J Clin Med. 2025 Jun 17;14(12):4309. doi: 10.3390/jcm14124309 (PMC12194356; doi:10.3390/jcm14124309)
Supplement: Supplementary file 1 [file jcm-14-04309-s001.zip › jcm-3598360-supplementary.pdf]

Supplementary material

Table S1. Characteristics of participating physicians treating patients with HES or EGPA.

**Physicians treating patients with HES (N=121)**	**France n=25**	**Germany n=23**	**Italy n=26**	**Spain n=25**	**UK n=22**
**Physician specialty,** n (%)					
Allergy	5 (20.0)	3 (13.0)	4 (15.4)	6 (24.0)	2 (9.1)
Immunology	1 (4.0)	0 (0.0)	3 (11.5)	2 (8.0)	0 (0.0)
Rheumatology	1 (4.0)	1 (4.3)	7 (26.9)	4 (16.0)	2 (9.1)
Pulmonology	0 (0.0)	5 (21.7)	3 (11.5)	0 (0.0)	1 (4.5)
Hematology	13 (52.0)	11 (47.8)	4 (15.4)	6 (24.0)	17 (77.3)
Internal medicine	5 (20.0)	3 (13.0)	5 (19.2)	7 (28.0)	0 (0.0)
**Primary practice setting,*** n (%)					
Academic-based	17 (68.0)	14 (60.9)	16 (61.5)	22 (88.0)	19 (86.4)
Community-based	9 (36.0)	9 (39.1)	10 (38.5)	3 (12.0)	4 (18.2)
**Practice size,** n (%)					
**Physicians treating patients with HES (N=121)**	**France n=25**	**Germany n=23**	**Italy n=26**	**Spain n=25**	**UK n=22**
Solo (1 physician)	0 (0.0)	3 (13.0)	1 (3.8)	0 (0.0)	0 (0.0)
Small (2–10 physicians)	1 (4.0)	8 (34.8)	5 (19.2)	3 (12.0)	4 (18.2)
Medium (11–50 physicians)	6 (24.0)	9 (39.1)	8 (30.8)	4 (16.0)	8 (36.4)
Large (≥51 physicians)	18 (72.0)	3 (13.0)	12 (46.2)	18 (72.0)	10 (45.5)
**Number of years in practice,** n (%)					
1–5 years	0 (0.0)	0 (0.0)	2 (7.7)	0 (0.0)	0 (0.0)
6–10 years	2 (8.0)	2 (8.7)	1 (3.8)	3 (12.0)	1 (4.5)

11–20 years	17 (68.0)	21 (91.3)	10 (38.5)	12 (48.0)	10 (45.5)
>20 years	6 (24.0)	0 (0.0)	13 (50.0)	10 (40.0)	11 (50.0)
**Number patients with HES in the past 12 months per physician,** median (IQR)	20.0 (8.0, 70.0)	12.0 (5.0, 18.0)	17.5 (8.0, 50.0)	11.0 (8.0, 50.0)	11.0 (5.0, 22.0)
**Number of charts completed per physician,** median (IQR)	2.0 (2.0, 2.0)	2.0 (2.0, 2.0)	2.0 (1.0, 2.0)	2.0 (2.0, 2.0)	2.0 (2.0, 3.0)
**Physicians treating patients with EGPA (N=204)**	**France n=41**	**Germany n=39**	**Italy n=41**	**Spain n=38**	**UK n=45**
**Physician specialty,** n (%)					
Allergy	2 (4.9)	5 (12.8)	5 (12.2)	11 (28.9)	3 (6.7)
Immunology	2 (4.9)	0 (0.0)	9 (22.0)	2 (5.3)	0 (0.0)
Rheumatology	9 (22.0)	20 (51.3)	16 (39.0)	15 (39.5)	29 (64.4)
Pulmonology	28 (68.3)	14 (35.9)	11 (26.8)	10 (26.3)	13 (28.9)
**Primary practice setting,*** n (%)					
Academic-based	17 (41.5)	26 (66.7)	27 (65.9)	36 (94.7)	28 (62.2)
Community-based	24 (58.5)	13 (33.3)	17 (41.5)	2 (5.3)	17 (37.8)
**Practice size,** n (%)					
Solo (1 physician)	2 (4.9)	3 (7.7)	1 (2.4)	0 (0.0)	0 (0.0)
Small (2–10 physicians)	9 (22.0)	14 (35.9)	11 (26.8)	6 (15.8)	11 (24.4)
Medium (11–50 physicians)	6 (14.6)	13 (33.3)	7 (17.1)	3 (7.9)	18 (40.0)
Large (≥51 physicians)	24 (58.5)	9 (23.1)	22 (53.7)	29 (76.3)	16 (35.6)
**Physicians treating patients with EGPA (N=204)**	**France n=41**	**Germany n=39**	**Italy n=41**	**Spain n=38**	**UK n=45**
**Number of years in practice,** n (%)					
1–5 years	0 (0.0)	1 (2.6)	0 (0.0)	0 (0.0)	1 (2.2)

6–10 years	9 (22.0)	7 (17.9)	6 (14.6)	0 (0.0)	7 (15.6)
11–20 years	21 (51.2)	22 (56.4)	14 (34.1)	15 (39.5)	23 (51.1)
>20 years	11 (26.8)	9 (23.1)	21 (51.2)	23 (60.5)	14 (31.1)
**Number of patients with EGPA in the past 12 months per physician,** median (IQR)	10.0 (4.0, 20.0)	15.0 (6.0, 25.0)	20.0 (5.0, 40.0)	5.5 (4.0, 20.0)	10.0 (4.0, 14.0)
**Number of charts completed per physician,** median (IQR)	2.0 (1.0, 2.0)	2.0 (1.0, 2.0)	2.0 (1.0, 2.0)	2.0 (2.0, 2.0)	2.0 (1.0, 2.0)

*Physicians were allowed to select more than one practice setting.

EGPA, eosinophilic granulomatosis with polyangiitis; HES, hypereosinophilic syndrome; IQR, interquartile range; SD, standard deviation; UK, United Kingdom.

Table S2. Other treatment use in patients with HES and ongoing HES therapies at EOF.

**HES therapies by category* (N=280)**	**France n=61**	**Germany n=53**	**Italy n=52**	**Spain n=52**	**UK n=62**
**Other HES therapies used by ≥5% of patients in any country,**[†] n (%)	15 (24.6)	4 (7.5)	4 (7.7)	0 (0)	7 (11.3)
Immunoglobulin (intravenous)	5 (8.2)	2 (3.8)	4 (7.7)	0 (0)	3 (4.8)
Ivermectin	6 (9.8)	0 (0)	0 (0)	0 (0)	2 (3.2)
**Other treatments used related to the complications and adverse effects of immunosuppressive medications,**[‡] n (%)	21 (34.4)	31 (58.5)	31 (59.6)	25 (48.1)	37 (59.7)
Treatments for the improvement of bone mineral density[§]	11 (18)	17 (32.1)	21 (40.4)	15 (28.8)	19 (30.6)
Treatment for infections in relation to HES therapies[¶]	4 (6.6)	11 (20.8)	8 (15.4)	7 (13.5)	17 (27.4)
Thyroid hormone replacement treatments	1 (1.6)	9 (17)	4 (7.7)	1 (1.9)	8 (12.9)
Treatment for diabetes	4 (6.6)	6 (11.3)	4 (7.7)	2 (3.8)	12 (19.4)
Folic acid supplements	10 (16.4)	10 (18.9)	12 (23.1)	12 (23.1)	16 (25.8)
**Ongoing treatments received at EOF**[**††‡]					
**OCS, n (%)**	17 (27.9)	29 (54.7)	26 (50)	20 (38.5)	33 (53.2)
Prednisone or prednisolone	13 (21.3)	25 (47.2)	16 (30.8)	17 (32.7)	26 (41.9)
Methylprednisolone	1 (1.6)	2 (3.8)	11 (21.2)	3 (5.8)	5 (8.1)
Cortisone	3 (4.9)	2 (3.8)	0 (0)	0 (0)	2 (3.2)
**Immunosuppressants or cytotoxic agents, used by ≥5% of patients in any country,**[‡‡] n (%)	27 (44.3)	18 (34)	21 (40.4)	13 (25)	20 (32.3)
Azathioprine	4 (6.6)	3 (5.7)	4 (7.7)	2 (3.8)	4 (6.5)
Imatinib mesylate	15 (24.6)	7 (13.2)	9 (17.3)	4 (7.7)	3 (4.8)
Methotrexate	0 (0.0)	5 (9.4)	0 (0.0)	6 (11.5)	1 (1.6)

HES therapies by category* (N=280)	France n=61	Germany n=53	Italy n=52	Spain n=52	UK n=62
**Biologics,** n (%)	23 (37.7)	14 (26.4)	17 (32.7)	15 (28.8)	28 (45.2)
Mepolizumab	8 (13.1)	2 (3.8)	11 (21.2)	7 (13.5)	4 (6.5)
Alemtuzumab	1 (1.6)	1 (1.9)	2 (3.8)	0 (0)	11 (17.7)
Benralizumab	6 (9.8)	7 (13.2)	5 (9.6)	2 (3.8)	7 (11.3)
Dupilumab	4 (6.6)	3 (5.7)	1 (1.9)	0 (0)	6 (9.7)
Omalizumab	0 (0)	2 (3.8)	0 (0)	3 (5.8)	0 (0)
Reslizumab	5 (8.2)	1 (1.9)	0 (0)	2 (3.8)	1 (1.6)
Rituximab	4 (6.6)	4 (7.5)	0 (0)	4 (7.7)	4 (6.5)

*Treatment patterns for HES therapies were assessed between HES diagnosis and the EOF (i.e., last physician encounter or death). †Treatments used by <5% of patients in each country were: warfarin, stem cell transplant, other steroids, and albendazole. Stem cell transplant, other steroids, and albendazole were not listed in the case report form, but were reported as a free-text response by one or more physician respondents. ‡Assessed between index date and the EOF. §Examples of treatments for improvement of bone mineral density include alendronate, risedronate sodium, ibandronate, and zoledronic acid. ¶Examples of treatments for infections in relation to HES therapies include antitubercular agents, acyclovir, valacyclovir, and fluconazole. **Ongoing treatments are treatments that the patient was indicated as taking at EOF. ††When categorized as an ongoing HES treatment received at the EOF, immunoglobulin (intravenous), warfarin, and stem cell transplant were used by <5% of patients in each country. ‡‡Ongoing treatments used by <5% of patients in each country were: chlorambucil, cyclophosphamide, cyclosporine, etoposide, hydroxyurea, interferon-alpha, peg-interferon, ruxolitinib, and leflunomide.

EOF, end of follow-up; HES, hypereosinophilic syndrome; OCS, oral corticosteroid; UK, United Kingdom.

Table S3. Other treatment use in patients with EGPA and ongoing treatments at the EOF.

**EGPA therapies by category*(N=407)**	**France n=81**	**Germany n=80**	**Italy n=80**	**Spain n=85**	**UK n=81**
**Other treatments for the control of EGPA-related clinical manifestations, used by ≥5% of patients in any country,** [†] n (%)	54 (66.7)	50 (62.5)	73 (91.3)	69 (81.2)	56 (69.1)
Albuterol	30 (37.0)	5 (6.3)	27 (33.8)	43 (50.6)	14 (17.3)
Analgesics	23 (28.4)	19 (23.8)	16 (20.0)	22 (25.9)	16 (19.8)
Budesonide – formoterol	35 (43.2)	18 (22.5)	41 (51.3)	39 (45.9)	27 (33.3)
Ipratropium bromide	5 (6.2)	2 (2.5)	8 (10.0)	15 (17.6)	10 (12.3)
Montelukast	13 (16.0)	10 (12.5)	14 (17.5)	12 (14.1)	13 (16.0)
Ramipril	8 (9.9)	20 (25.0)	9 (11.3)	7 (8.2)	11 (13.6)
Tiotropium	15 (18.5)	6 (7.5)	9 (11.3)	7 (8.2)	8 (9.9)
Valsartan	6 (7.4)	6 (7.5)	9 (11.3)	2 (2.4)	4 (4.9)
**Other treatments used related to the complications and adverse effects of immunosuppressive medications, used by ≥5% of patients in any country,**[‡] n (%)	43 (53.1)	43 (53.8)	55 (68.8)	50 (58.8)	47 (58.0)
Treatments for the improvement of bone mineral density	28 (34.6)	28 (35.0)	37 (46.3)	37 (43.5)	33 (40.7)
Treatment for infections in relation to EGPA therapies	17 (21.0)	5 (6.3)	13 (16.3)	15 (17.6)	5 (6.2)
Treatments for recovering cytopenia	8 (9.9)	1 (1.3)	2 (2.5)	8 (9.4)	2 (2.5)
Treatments for hemorrhagic cystitis	5 (6.2)	0 (0.0)	4 (5.0)	2 (2.4)	7 (8.6)
Treatments for gastrointestinal toxicity	5 (6.2)	5 (6.3)	12 (15.0)	11 (12.9)	7 (8.6)

EGPA therapies by category*(N=407)	France n=81	Germany n=80	Italy n=80	Spain n=85	UK n=81
Thyroid hormone replacement treatments	4 (4.9)	5 (6.3)	1 (1.3)	9 (10.6)	3 (3.7)
Folic acid supplements	12 (14.8)	15 (18.8)	19 (23.8)	27 (31.8)	15 (18.5)
**Ongoing treatments received at EOF§**					
**OCS,** n (%)	54 (66.7)	50 (62.5)	53 (66.3)	51 (60.0)	46 (56.8)
Prednisone or prednisolone	50 (61.7)	48 (60.0)	40 (50.0)	42 (49.4)	45 (55.6)
Methylprednisolone	2 (2.5)	1 (1.3)	9 (11.3)	7 (8.2)	1 (1.2)
Cortisone	2 (2.5)	1 (1.3)	4 (5.0)	2 (2.4)	0 (0.0)
**Immunosuppressive agents and other therapies, used by ≥5% of patients in any country,¶** n (%)	25 (30.9)	43 (53.8)	32 (40.0)	36 (42.4)	47 (58.0)
Azathioprine	10 (12.3)	22 (27.5)	12 (15.0)	7 (8.2)	20 (24.7)
Cyclophosphamide	2 (2.5)	8 (10.0)	2 (2.5)	7 (8.2)	1 (1.2)
Methotrexate	9 (11.1)	7 (8.8)	11 (13.8)	11 (12.9)	10 (12.3)
Mycophenolate	1 (1.2)	1 (1.3)	2 (2.5)	5 (5.9)	16 (19.8)
**Biologics, used by ≥5% of patients in any country,**** n (%)	29 (35.8)	11 (13.8)	38 (47.5)	35 (41.2)	17 (21.0)
Mepolizumab	11 (13.6)	4 (5.0)	22 (27.5)	11 (12.9)	7 (8.6)
Benralizumab	3 (3.7)	3 (3.8)	6 (7.5)	9 (10.6)	1 (1.2)
Omalizumab	0 (0.0)	4 (5.0)	3 (3.8)	6 (7.1)	3 (3.7)
Reslizumab	3 (3.7)	0 (0.0)	2 (2.5)	6 (7.1)	1 (1.2)
Rituximab	15 (18.5)	2 (2.5)	6 (7.5)	9 (10.6)	7 (8.6)
**Other treatments for the control of EGPA-related clinical manifestations,** n (%)	49 (60.5)	46 (57.5)	62 (77.5)	59 (69.4)	55 (67.9)

[*]Treatment patterns for EGPA therapies were assessed between EGPA diagnosis and EOF. [†]Treatments used by <5% of patients in each country were: doxazosin, levalbuterol, theophylline, and zafirlukast. [‡]Treatments used by <5% of patients in each country were: treatments for hepatotoxicity and insulin. [§]Ongoing treatments are treatments that the

patient was indicated as taking at EOF. [¶]Treatments used by <5% of patients in each country were: cyclosporine, immunoglobulin (intravenous), interferon-alpha, leflunomide, and plasma exchange. **Treatments used by <5% of patients in each country were dupilumab.

EGPA, eosinophilic granulomatosis with polyangiitis; EOF, end of follow-up; OCS, oral corticosteroid; UK, United Kingdom.

Table S4. Clinical manifestations and symptom severity in patients with HES.

**Clinical manifestations* in patients with HES (N=280)**	**France n=61**	**Germany n=53**	**Italy n=52**	**Spain n=52**	**UK n=62**
**Number of distinct clinical manifestations, continuous**					
Mean (SD)	3.3 (3.2)	3.3 (3.0)	4.6 (4.7)	4.7 (3.7)	3 (3.5)
Median (IQR)	3 (1.0, 4.0)	3 (1.0, 5.0)	4 (2.0, 6.0)	4 (2.0, 6.5)	2 (1.0, 4.0)
**Number of distinct clinical manifestations, categorical,** n (%)					
0	8 (13.1)	7 (13.2)	10 (19.2)	6 (11.5)	13 (21.0)
1–2	18 (29.5)	18 (34.0)	13 (25.0)	9 (17.3)	24 (38.7)
3–4	22 (36.1)	14 (26.4)	11 (21.2)	16 (30.8)	10 (16.1)
5–6	9 (14.8)	6 (11.3)	6 (11.5)	8 (15.4)	9 (14.5)
≥7	4 (6.6)	8 (15.1)	12 (23.1)	13 (25.0)	6 (9.7)
**Clinical manifestations by organ involvement,*** n (%)					
**Constitutional**	**46 (75.4)**	**31 (58.5)**	**27 (51.9)**	**41 (78.8)**	**32 (51.6)**
Fatigue	42 (68.9)	21 (39.6)	22 (42.3)	32 (61.5)	22 (35.5)
Proportion of manifestations classified as moderate to severe†	39 (92.9)	14 (66.7)	12 (54.5)	25 (78.1)	18 (81.8)
Pain	18 (29.5)	12 (22.6)	13 (25.0)	24 (46.2)	12 (19.4)
Proportion of manifestations classified as moderate to severe†	15 (83.3)	9 (75.0)	11 (84.6)	23 (95.8)	5 (41.7)
Chills/sweats	7 (11.5)	4 (7.5)	7 (13.5)	12 (23.1)	7 (11.3)
Proportion of manifestations classified as moderate to severe†	5 (71.4)	3 (75.0)	5 (71.4)	10 (83.3)	6 (85.7)
Angioedema	1 (1.6)	6 (11.3)	6 (11.5)	9 (17.3)	4 (6.5)
Proportion of manifestations classified as moderate to severe†	0 (0.0)	3 (50.0)	4 (66.7)	6 (66.7)	4 (100.0)
**Lung**	**28 (45.9)**	**28 (52.8)**	**29 (55.8)**	**25 (48.1)**	**27 (43.5)**
Asthma	4 (6.6)	16 (30.2)	22 (42.3)	15 (28.8)	9 (14.5)

Clinical manifestations* in patients with HES (N=280)	France n=61	Germany n=53	Italy n=52	Spain n=52	UK n=62
Proportion of manifestations classified as moderate to severe†	4 (100.0)	9 (56.3)	17 (77.3)	13 (86.7)	4 (44.4)
Dyspnea (shortness of breath)	9 (14.8)	12 (22.6)	15 (28.8)	8 (15.4)	8 (12.9)
Proportion of manifestations classified as moderate to severe†	6 (66.7)	6 (50.0)	9 (60.0)	6 (75.0)	6 (75.0)
Coughing	9 (14.8)	7 (13.2)	13 (25.0)	6 (11.5)	7 (11.3)
Proportion of manifestations classified as moderate to severe†	7 (77.8)	2 (28.6)	11 (84.6)	5 (83.3)	5 (71.4)
Pulmonary infiltration	16 (26.2)	4 (7.5)	5 (9.6)	4 (7.7)	6 (9.7)
Proportion of manifestations classified as moderate to severe†	13 (81.3)	3 (75.0)	3 (60.0)	3 (75.0)	5 (83.3)
Wheezing	4 (6.6)	1 (1.9)	13 (25.0)	4 (7.7)	8 (12.9)
Proportion of manifestations classified as moderate to severe†	1 (25.0)	0 (0.0)	12 (92.3)	3 (75.0)	2 (25.0)
**Skin**	**23 (37.7)**	**20 (37.7)**	**25 (48.1)**	**32 (61.5)**	**35 (56.5)**
Itch	17 (27.9)	12 (22.6)	20 (38.5)	23 (44.2)	23 (37.1)
Proportion of manifestations classified as moderate to severe†	14 (82.4)	8 (66.7)	9 (45.0)	18 (78.3)	13 (56.5)
Rash	10 (16.4)	11 (20.8)	7 (13.5)	22 (42.3)	14 (22.6)
Proportion of manifestations classified as moderate to severe†	9 (90.0)	6 (54.5)	1 (14.3)	16 (72.7)	12 (85.7)
Hives/urticaria	4 (6.6)	7 (13.2)	7 (13.5)	6 (11.5)	8 (12.9)
Proportion of manifestations classified as moderate to severe†	3 (75.0)	4 (57.1)	5 (71.4)	4 (66.7)	8 (100.0)
**ENT**	**12 (19.7)**	**12 (22.6)**	**20 (38.5)**	**20 (38.5)**	**18 (29.0)**
Nasal congestion	6 (9.8)	7 (13.2)	15 (28.8)	13 (25.0)	5 (8.1)
Proportion of manifestations classified as moderate to severe†	5 (83.3)	6 (85.7)	8 (53.3)	8 (61.5)	2 (40.0)
Sinus headache/facial pain/pressure	3 (4.9)	6 (11.3)	9 (17.3)	7 (13.5)	5 (8.1)
Proportion of manifestations classified as moderate to severe†	1 (33.3)	5 (83.3)	7 (77.8)	4 (57.1)	5 (100.0)
Postnasal drip	4 (6.6)	0 (0.0)	10 (19.2)	6 (11.5)	8 (12.9)
Proportion of manifestations classified as moderate to severe†	3 (75.0)	0 (0.0)	6 (60.0)	3 (50.0)	4 (50.0)

Clinical manifestations* in patients with HES (N=280)	France n=61	Germany n=53	Italy n=52	Spain n=52	UK n=62
Purulent rhinorrhea	3 (4.9)	1 (1.9)	5 (9.6)	1 (1.9)	1 (1.6)
Proportion of manifestations classified as moderate to severe†	1 (33.3)	0 (0.0)	3 (60.0)	0 (0.0)	1 (100.0)
Ear fullness	2 (3.3)	1 (1.9)	2 (3.8)	1 (1.9)	5 (8.1)
Proportion of manifestations classified as moderate to severe†	1 (50.0)	1 (100.0)	0 (0.0)	0 (0.0)	5 (100.0)
**Gastrointestinal**	**9 (14.8)**	**19 (35.8)**	**16 (30.8)**	**18 (34.6)**	**11 (17.7)**
Diarrhea	6 (9.8)	6 (11.3)	10 (19.2)	9 (17.3)	5 (8.1)
Proportion of manifestations classified as moderate to severe†	3 (50.0)	3 (50.0)	7 (70.0)	3 (33.3)	4 (80.0)
Abdominal pain	3 (4.9)	7 (13.2)	4 (7.7)	11 (21.2)	7 (11.3)
Proportion of manifestations classified as moderate to severe†	2 (66.7)	4 (57.1)	3 (75.0)	4 (36.4)	3 (42.9)
Nausea/vomiting	3 (4.9)	11 (20.8)	7 (13.5)	4 (7.7)	3 (4.8)
Proportion of manifestations classified as moderate to severe†	2 (66.7)	7 (63.6)	1 (14.3)	2 (50.0)	2 (66.7)
Difficulty in swallowing food	2 (3.3)	1 (1.9)	4 (7.7)	2 (3.8)	3 (4.8)
Proportion of manifestations classified as moderate to severe†	0 (0.0)	0 (0.0)	3 (75.0)	1 (50.0)	1 (33.3)
**Neuropsychiatric**	**7 (11.5)**	**12 (22.6)**	**7 (13.5)**	**5 (9.6)**	**7 (11.3)**
Sensory neuropathy	6 (9.8)	10 (18.9)	7 (13.5)	2 (3.8)	4 (6.5)
Proportion of manifestations classified as moderate to severe†	3 (50.0)	5 (50.0)	4 (57.1)	0 (0.0)	2 (50.0)
Motor neuropathy	2 (3.3)	1 (1.9)	2 (3.8)	2 (3.8)	2 (3.2)
Proportion of manifestations classified as moderate to severe†	1 (50.0)	1 (100.0)	0 (0.0)	0 (0.0)	1 (50.0)
Cognitive and mental status change	2 (3.3)	1 (1.9)	1 (1.9)	2 (3.8)	2 (3.2)
Proportion of manifestations classified as moderate to severe†	0 (0.0)	0 (0.0)	0 (0.0)	1 (50.0)	0 (0.0)
**Cardiovascular**	**9 (14.8)**	**7 (13.2)**	**7 (13.5)**	**10 (19.2)**	**4 (6.5)**
Cardiomyopathy	6 (9.8)	2 (3.8)	4 (7.7)	6 (11.5)	2 (3.2)
Proportion of manifestations classified as moderate to severe†	4 (66.7)	1 (50.0)	0 (0.0)	4 (66.7)	0 (0.0)

Clinical manifestations* in patients with HES (N=280)	France n=61	Germany n=53	Italy n=52	Spain n=52	UK n=62
Heart failure	3 (4.9)	5 (9.4)	3 (5.8)	2 (3.8)	1 (1.6)
Proportion with functional capacity					
Class I‡	3 (100.0)	5 (100.0)	3 (100.0)	2 (100.0)	1 (100.0)
Proportion with objective assessment					
Class A‡	3 (100.0)	5 (100.0)	3 (100.0)	2 (100.0)	1 (100.0)
Thromboembolism	1 (1.6)	2 (3.8)	3 (5.8)	2 (3.8)	2 (3.2)
Proportion of manifestations classified as moderate to severe†	0 (0.0)	2 (100.0)	1 (33.3)	0 (0.0)	1 (50.0)
Ischemic heart disease	1 (1.6)	1 (1.9)	2 (3.8)	2 (3.8)	1 (1.6)
Proportion of manifestations classified as moderate to severe†	0 (0.0)	1 (100.0)	1 (50.0)	1 (50.0)	0 (0.0)
Valvular disease	1 (1.6)	0 (0.0)	1 (1.9)	1 (1.9)	1 (1.6)
Proportion of manifestations classified as moderate to severe†	0 (0.0)	0 (0.0)	0 (0.0)	0 (0.0)	1 (100.0)
**Other**					
Kidney failure	1 (1.6)	0 (0.0)	0 (0.0)	1 (1.9)	0 (0.0)
Proportion of manifestations classified as moderate to severe†	1 (100)	0 (0.0)	0 (0.0)	1 (100)	0 (0.0)
**Biopsy confirmed tissue eosinophilia**	**3 (4.9)**	**1 (1.9)**	**2 (3.8)**	**3 (5.8)**	**3 (4.8)**
Proportion of manifestations classified as moderate to severe†	1 (33.3)	0 (0.0)	1 (50.0)	2 (66.7)	3 (100.0)

*Clinical manifestations were assessed between index date and EOF. †Severity was documented at first occurrence of a manifestation on the following scale: mild (present but minimal impact), moderate (significant impact on daily activities), severe (incapacitating). Moderate and severe severity have been combined (mild not shown). ‡Severity of heart failure was defined by functional capacity (Class I to IV; where I is no limitation on physical activity and IV is symptoms of heart failure at rest and increasing discomfort with physical activity) and objective assessment (Class A to D; where A is no evidence of cardiovascular disease and D is severe cardiovascular disease) based on NYHA heart failure criteria.

ENT, ear, nose, and throat; EOF, end of follow-up; HES, hypereosinophilic syndrome; IQR, interquartile range; NYHA, New York Heart Association; SD, standard deviation; UK, United Kingdom.

Table S5. Clinical manifestations and symptom severity in patients with EGPA.

Clinical manifestations* in patients with EGPA (N=407)	France n=81	Germany n=80	Italy n=80	Spain n=85	UK n=81
**Number of distinct clinical manifestations, continuous**					
Mean (SD)	4.1 (4.0)	3.2 (3.5)	4.3 (4.1)	4.5 (3.6)	4.5 (4.6)
Median (IQR)	3 (1.0, 5.0)	2 (1.0, 5.0)	3 (1.0, 6.0)	4 (2.0, 6.0)	4 (1.0, 6.0)
**Number of distinct clinical manifestations, categorical, n (%)**					
0	12 (14.8)	14 (17.5)	15 (18.8)	7 (8.2)	14 (17.3)
1–2	22 (27.2)	29 (36.3)	18 (22.5)	20 (23.5)	15 (18.5)
3–5	27 (33.3)	19 (23.8)	24 (30)	32 (37.6)	31 (38.3)
6–8	10 (12.3)	14 (17.5)	9 (11.3)	16 (18.8)	12 (14.8)
9–12	6 (7.4)	1 (1.3)	10 (12.5)	7 (8.2)	4 (4.9)
13 and over	4 (4.9)	3 (3.8)	4 (5)	3 (3.5)	5 (6.2)
**Clinical manifestations by organ involvement, n (%)**					
**Lung**	**55 (67.9)**	**33 (41.3)**	**41 (51.3)**	**57 (67.1)**	**41 (50.6)**
Shortness of breath	27 (33.3)	25 (31.3)	27 (33.8)	39 (45.9)	33 (40.7)
Proportion of manifestations classified as moderate to severe†	21 (77.8)	21 (84.0)	21 (77.8)	33 (84.6)	21 (63.7)
Lung infiltrates	30 (37.0)	12 (15.0)	23 (28.8)	16 (18.8)	24 (29.6)
Proportion of manifestations classified as moderate to severe†	23 (76.7)	7 (58.3)	18 (78.2)	14 (87.6)	12 (50.0)
Severe asthma	32 (39.5)	10 (12.5)	22 (27.5)	31 (36.5)	6 (7.4)
Proportion of manifestations classified as moderate to severe†	27 (84.4)	8 (80.0)	20 (91.0)	31 (100.0)	5 (83.3)
Pleural effusion	2 (2.5)	5 (6.3)	1 (1.3)	7 (8.2)	6 (7.4)
Proportion of manifestations classified as moderate to severe†	1 (50.0)	4 (80.0)	1 (100.0)	6 (85.7)	4 (66.7)
Alveolar hemorrhage	4 (4.9)	1 (1.3)	1 (1.3)	3 (3.5)	6 (7.4)
Proportion of manifestations classified as moderate to severe†	1 (25.0)	0 (0.0)	1 (100.0)	3 (100.0)	4 (66.7)

Clinical manifestations* in patients with EGPA (N=407)	France n=81	Germany n=80	Italy n=80	Spain n=85	UK n=81
**ENT**	**42 (51.9)**	**35 (43.8)**	**43 (53.8)**	**52 (61.2)**	**45 (55.6)**
Allergic rhinitis	28 (34.6)	24 (30.0)	23 (28.8)	37 (43.5)	28 (34.6)
Proportion of manifestations classified as moderate to severe†	19 (67.9)	16 (66.7)	17 (73.9)	27 (73.0)	23 (82.2)
Paranasal sinusitis	13 (16.0)	19 (23.8)	18 (22.5)	21 (24.7)	19 (23.5)
Proportion of manifestations classified as moderate to severe†	11 (84.7)	12 (63.2)	13 (72.3)	15 (71.4)	12 (63.2)
Nasal polyposis	18 (22.2)	10 (12.5)	25 (31.3)	20 (23.5)	16 (19.8)
Proportion of manifestations classified as moderate to severe†	12 (66.6)	5 (50.0)	21 (84.0)	17 (85.0)	11 (68.8)
Otitis media	3 (3.7)	1 (1.3)	1 (1.3)	3 (3.5)	6 (7.4)
Proportion of manifestations classified as moderate to severe†	2 (66.7)	0 (0.0)	0 (0.0)	2 (66.7)	4 (66.6)
**Constitutional**	**35 (43.2)**	**38 (47.5)**	**36 (45.0)**	**44 (51.8)**	**45 (55.6)**
Fatigue	33 (40.7)	33 (41.3)	32 (40.0)	35 (41.2)	41 (50.6)
Proportion of manifestations classified as moderate to severe†	26 (78.8)	18 (54.6)	27 (84.4)	25 (71.5)	36 (87.8)
Myalgia/arthralgia	19 (23.5)	14 (17.5)	24 (30.0)	28 (32.9)	26 (32.1)
Proportion of manifestations classified as moderate to severe†	9 (47.4)	9 (64.2)	19 (79.2)	21 (75.0)	18 (69.2)
**Skin**	**28 (34.6)**	**30 (37.5)**	**38 (47.5)**	**43 (50.6)**	**31 (38.3)**
Itch	11 (13.6)	18 (22.5)	18 (22.5)	19 (22.4)	12 (14.8)
Proportion of manifestations classified as moderate to severe†	3 (27.3)	15 (83.4)	14 (77.8)	12 (63.1)	7 (58.4)
Urticaria	13 (16.0)	15 (18.8)	17 (21.3)	17 (20.0)	12 (14.8)
Proportion of manifestations classified as moderate to severe†	8 (61.6)	9 (60.0)	13 (76.5)	12 (70.5)	8 (66.6)
Purpura	11 (13.6)	7 (8.8)	9 (11.3)	20 (23.5)	18 (22.2)
Proportion of manifestations classified as moderate to severe†	7 (63.6)	5 (71.4)	6 (66.7)	15 (75.0)	13 (72.2)
Ulcers	4 (4.9)	2 (2.5)	13 (16.3)	3 (3.5)	5 (6.2)
Proportion of manifestations classified as moderate to severe†	3 (75.0)	2 (100.0)	13 (100.0)	2 (66.7)	3 (60.0)
**Gastrointestinal**	**17 (21.0)**	**17 (21.3)**	**17 (21.3)**	**13 (15.3)**	**15 (18.5)**

Clinical manifestations* in patients with EGPA (N=407)	France n=81	Germany n=80	Italy n=80	Spain n=85	UK n=81
Abdominal pain	12 (14.8)	11 (13.8)	5 (6.3)	6 (7.1)	9 (11.1)
Proportion of manifestations classified as moderate to severe†	5 (41.7)	8 (72.7)	3 (60.0)	4 (66.7)	6 (66.6)
Diarrhea	4 (4.9)	5 (6.3)	4 (5.0)	8 (9.4)	10 (12.3)
Proportion of manifestations classified as moderate to severe†	1 (25.0)	3 (60.0)	3 (75.0)	4 (50.0)	3 (30.0)
Nausea/vomiting	6 (7.4)	3 (3.8)	5 (6.3)	5 (5.9)	6 (7.4)
Proportion of manifestations classified as moderate to severe†	2 (33.3)	1 (33.3)	3 (60.0)	3 (60.0)	4 (66.7)
Gastrointestinal bleeding	2 (2.5)	3 (3.8)	7 (8.8)	1 (1.2)	4 (4.9)
Proportion of manifestations classified as moderate to severe†	0 (0.0)	3 (100.0)	6 (85.7)	0 (0.0)	1 (25.0)
**Renal**	**16 (19.8)**	**13 (16.3)**	**19 (23.8)**	**10 (11.8)**	**20 (24.7)**
Glomerulonephritis	10 (12.3)	4 (5.0)	12 (15.0)	5 (5.9)	12 (14.8)
Proportion of manifestations classified as moderate to severe†	5 (50.0)	3 (75.0)	10 (83.3)	3 (60.0)	5 (41.6)
Proteinuria	9 (11.1)	9 (11.3)	7 (8.8)	4 (4.7)	10 (12.3)
Proportion of manifestations classified as moderate to severe†	3 (33.3)	3 (33.3)	5 (71.4)	3 (75.0)	6 (60.0)
Hematuria	1 (1.2)	3 (3.8)	5 (6.3)	6 (7.1)	7 (8.6)
Proportion of manifestations classified as moderate to severe†	1 (100.0)	2 (66.7)	5 (100.0)	2 (33.3)	4 (57.1)
**Cardiovascular**	**7 (8.6)**	**6 (7.5)**	**14 (17.5)**	**11 (12.9)**	**7 (8.6)**
Cardiac arrhythmia	4 (4.9)	3 (3.8)	3 (3.8)	5 (5.9)	3 (3.7)
Proportion of manifestations classified as moderate to severe†	3 (75.0)	1 (33.3)	2 (66.7)	2 (40.0)	1 (33.3)
Ischemic heart disease	1 (1.2)	2 (2.5)	7 (8.8)	1 (1.2)	3 (3.7)
Proportion of manifestations classified as moderate to severe†	0 (0.0)	1 (50.0)	7 (100.0)	0 (0.0)	2 (66.7)
Cardiomyopathy	2 (2.5)	2 (2.5)	1 (1.3)	2 (2.4)	2 (2.5)
Proportion of manifestations classified as moderate to severe†	1 (50.0)	0 (0.0)	1 (100.0)	2 (100.0)	1 (50.0)
Peripheral vascular disease	2 (2.5)	0 (0.0)	3 (3.8)	3 (3.5)	1 (1.2)
Proportion of manifestations classified as moderate to severe†	1 (50.0)	0 (0.0)	2 (66.6)	1 (33.3)	0 (0.0)

Clinical manifestations* in patients with EGPA (N=407)	France n=81	Germany n=80	Italy n=80	Spain n=85	UK n=81
Valvular disease	2 (2.5)	1 (1.3)	0 (0.0)	4 (4.7)	2 (2.5)
Proportion of manifestations classified a moderate to severe†	2 (100.0)	0 (0.0)	0 (0.0)	2 (50.0)	0 (0.0)
Pericarditis	0 (0.0)	2 (2.5)	3 (3.8)	2 (2.4)	1 (1.2)
Proportion of manifestations classified as moderate to severe†	0 (0.0)	2 (100.0)	2 (66.7)	1 (50.0)	1 (100.0)
Heart failure	2 (2.5)	1 (1.3)	0 (0.0)	1 (1.2)	3 (3.7)
Proportion with functional capacity classification‡					
Class II	2 (100.0)	0 (0.0)	N/A	1 (100.0)	1 (33.3)
Class III	0 (0.0)	1 (100.0)	N/A	0 (0.0)	1 (33.3)
Proportion with objective assessment classification‡					
Class A	1 (50.0)	0 (0.0)	N/A	0 (0.0)	0 (0.0)
Class B	1 (50.0)	0 (0.0)	N/A	1 (100.0)	1 (33.3)
Class C	0 (0.0)	1 (100.0)	N/A	0 (0.0)	1 (33.3)
**Neuropsychiatric**	**11 (13.6)**	**10 (12.5)**	**18 (22.5)**	**16 (18.8)**	**20 (24.7)**
Peripheral neuropathy	8 (9.9)	7 (8.8)	14 (17.5)	13 (15.3)	9 (11.1)
Proportion of manifestations classified as moderate to severe†	5 (62.5)	5 (71.4)	10 (71.4)	7 (53.9)	6 (66.6)
Mononeuritis	3 (3.7)	3 (3.8)	3 (3.8)	3 (3.5)	10 (12.3)
Proportion of manifestations classified as moderate to severe†	3 (100.0)	2 (66.6)	2 (66.7)	3 (100.0)	7 (70.0)
Cranial nerve palsies or involvement	1 (1.2)	1 (1.3)	0 (0.0)	1 (1.2)	3 (3.7)
Proportion of manifestations classified as moderate to severe†	1 (100.0)	0 (0.0)	0 (0.0)	1 (100.0)	0 (0.0)
Psychosis	0 (0.0)	0 (0.0)	2 (2.5)	1 (1.2)	2 (2.5)
Proportion of manifestations classified as moderate to severe†	0 (0.0)	0 (0.0)	0 (0.0)	0 (0.0)	0 (0.0)
Stroke	2 (2.5)	0 (0.0)	0 (0.0)	0 (0.0)	3 (3.7)
Proportion of manifestations classified as moderate to severe†	1 (50.0)	0 (0.0)	0 (0.0)	0 (0.0)	1 (33.3)

Clinical manifestations* in patients with EGPA (N=407)	France n=81	Germany n=80	Italy n=80	Spain n=85	UK n=81
**Biopsy confirmed eosinophilic vasculitis or eosinophilic inflammation**	16 (19.8)	2 (2.5)	7 (8.8)	10 (11.8)	7 (8.6)
Proportion of manifestations classified as moderate to severe†	10 (62.5)	1 (50.0)	6 (85.7)	7 (70.0)	7 (100.0)

*Clinical manifestations were assessed between index date and the EOF. †Severity was documents at first occurrence of a manifestation on the following scale: mild (present but minimal impact), moderate (significant impact on daily activities), severe (incapacitating). Moderate and severe severity have been combined (mild not shown). ‡Severity of heart failure was defined by functional capacity (Class I to IV; where I is no limitation on physical activity and IV is symptoms of heart failure at rest and increasing discomfort with physical activity) and objective assessment (Class A to D; where A is no evidence of cardiovascular disease and D is severe cardiovascular disease) based on NYHA heart failure criteria. Severity classification was unknown for 1 case from Italy.

EGPA, eosinophilic granulomatosis with polyangiitis; ENT, ear, nose, and throat; EOF, end of follow-up; IQR, interquartile range; N/A, not available; UK, United Kingdom.

Table S6. HCRU in HES.

HCRU among patients with HES (N=280)	France n=61	Germany n=53	Italy n=52	Spain n=52	UK n=62
**Proportion of patients with any HES-related visits,*** n (%)					
**Hospitalizations**	20 (32.8)	15 (28.3)	17 (32.7)	6 (11.5)	27 (43.5)
Average length of stay per hospitalization, days					
Mean (SD)	9.9 (7.3)	12.3 (13.1)	11.5 (5.9)	15.3 (4.2)	9.9 (11.0)
Median (IQR)	10 (4.0, 15.0)	7 (4.0, 21.0)	10 (7.0, 15.0)	14 (12.0, 20.0)	7 (5.0, 13.0)
**ER visits**	18 (29.5)	11 (20.8)	7 (13.5)	13 (25.0)	23 (37.1)
**Outpatient visits**	57 (93.4)	43 (81.1)	41 (78.8)	47 (90.4)	55 (88.7)
**Number of EGPA-related visits, per person per year***†					
**Hospitalizations**					
Mean (SD)	0.4 (1.3)	0.6 (1.9)	0.3 (0.6)	0.1 (0.2)	0.5 (1.0)
Median (IQR)	0.0 (0.0, 0.5)	0.0 (0.0, 0.3)	0.0 (0.0, 0.4)	0.0 (0.0, 0.0)	0.0 (0.0, 0.6)
**ER visits**					
Mean (SD)	0.5 (1.2)	0.2 (0.6)	0.2 (0.6)	0.2 (0.4)	0.4 (0.7)
Median (IQR)	0.0 (0.0, 0.4)	0.0 (0.0, 0.0)	0.0 (0.0, 0.0)	0.0 (0.0, 0.1)	0.0 (0.0, 0.6)
**Outpatient visits**					
**Overall outpatient visits**					
Mean (SD)	3.8 (3.1)	5.6 (8.0)	3.2 (3.0)	5.1 (4.9)	3.8 (3.5)
Median (IQR)	3.0 (1.5, 5.3)	4.1 (1.2, 6.2)	2.7 (0.6, 4.9)	4.2 (2.2, 6.2)	2.4 (1.3, 5.8)
**Unscheduled outpatient visits**					
Mean (SD)	0.6 (0.7)	2.0 (7.1)	0.6 (0.7)	1.0 (1.1)	1.1 (1.4)
Median (IQR)	0.4 (0.0, 0.9)	0.7 (0.0, 1.2)	0.5 (0.0, 1.0)	0.6 (0.0, 1.5)	0.6 (0.0, 1.5)

HCRU among patients with HES (N=280)	France n=61	Germany n=53	Italy n=52	Spain n=52	UK n=62
**Proportion of patients with any occurrence of tests related to the complications and monitoring of adverse effects of using immunosuppressive medications,*** n (%)					
**Bone mineral density testing**	24 (39.3)	27 (50.9)	23 (44.2)	17 (32.7)	24 (38.7)
**Cataract removal**	4 (6.6)	0 (0)	5 (9.6)	3 (5.8)	5 (8.1)
**Imaging tests (except simple X-rays)**					
CT scans	22 (36.1)	15 (28.3)	22 (42.3)	22 (42.3)	14 (22.6)
Chest radiographs	14 (23.0)	7 (13.2)	9 (17.3)	17 (32.7)	17 (27.4)
Echocardiograms or other cardiograms	21 (34.4)	13 (24.5)	20 (38.5)	20 (38.5)	18 (29.0)
Cardiac MRIs	13 (21.3)	5 (9.4)	6 (11.5)	5 (9.6)	11 (17.7)
Other imaging tests	3 (4.9)	2 (3.8)	4 (7.7)	0 (0)	6 (9.7)
**Number of tests related to the complications and monitoring of adverse effects of using immunosuppressive medications (per person per year)*†**					
**Bone mineral density testing**					
Mean (SD)	0.3 (0.5)	0.4 (0.5)	0.5 (0.8)	0.2 (0.3)	0.3 (0.5)
Median (IQR)	0 (0.0, 0.5)	0.3 (0.0, 0.6)	0 (0.0, 0.7)	0 (0.0, 0.3)	0 (0.0, 0.6)
**Cataract removal**					
Mean (SD)	0 (0.1)	0 (0)	0.1 (0.2)	0 (0.2)	0 (0.1)
Median (IQR)	0 (0.0, 0.0)	0 (0.0, 0.0)	0 (0.0, 0.0)	0 (0.0, 0.0)	0 (0.0, 0.0)
**Imaging tests (except simple X-rays)**					
**CT scans**					
Mean (SD)	0.6 (1.6)	0.2 (0.3)	0.3 (0.5)	0.5 (0.6)	0.2 (0.7)

HCRU among patients with HES (N=280)	France<br>n=61	Germany<br>n=53	Italy<br>n=52	Spain<br>n=52	UK<br>n=62
Median (IQR)	0 (0.0, 0.8)	0 (0.0, 0.3)	0 (0.0, 0.5)	0 (0.0, 0.9)	0 (0.0, 0.0)
**Chest radiographs**					
Mean (SD)	0.8 (3.2)	0.1 (0.4)	0.1 (0.4)	0.4 (0.7)	0.5 (1.2)
Median (IQR)	0 (0.0, 0.0)	0 (0.0, 0.0)	0 (0.0, 0.0)	0 (0.0, 0.5)	0 (0.0, 0.7)
**Echocardiograms or other cardiograms**					
Mean (SD)	0.7 (2.1)	0.3 (0.6)	0.3 (0.4)	0.2 (0.3)	0.3 (0.5)
Median (IQR)	0 (0.0, 0.8)	0 (0.0, 0.0)	0 (0.0, 0.6)	0 (0.0, 0.5)	0 (0.0, 0.6)
**Cardiac MRIs**					
Mean (SD)	0.2 (0.6)	0 (0.1)	0 (0.1)	0 (0.1)	0.1 (0.3)
Median (IQR)	0 (0.0, 0.0)	0 (0.0, 0.0)	0 (0.0, 0.0)	0 (0.0, 0.0)	0 (0.0, 0.0)
**Other imaging tests**					
Mean (SD)	0.1 (0.8)	0 (0.1)	0.1 (0.3)	0 (0.0)	0.1 (0.4)
Median (IQR)	0 (0.0, 0.0)	0 (0.0, 0.0)	0 (0.0, 0.0)	0 (0.0, 0.0)	0 (0.0, 0.0)

*HES-related visits and tests were assessed between index date and EOF. †The number of hospitalizations, ER visits, outpatient visits, and various types of tests were annualized.

CT, computed tomography; EOF, end of follow-up; ER, emergency room; HCRU, healthcare resource utilization; HES, hypereosinophilic syndrome; IQR, interquartile range; MRI, magnetic resonance imaging; SD, standard deviation; UK, United Kingdom.

Table S7. HCRU in EGPA.

HCRU among patients with EGPA (N=407)	France n=81	Germany n=80	Italy n=80	Spain n=85	UK n=81
**Proportion of patients with any EGPA-related visits,*** n (%)					
**Hospitalizations**	32 (39.5)	19 (23.8)	33 (41.3)	39 (45.9)	26 (32.1)
Average length of stay per hospitalization, days					
Mean (SD)	7.9 (8.6)	6.2 (2.8)	8.9 (7.1)	11.1 (6.2)	6.3 (4.0)
Median (IQR)	6.5 (4.0, 7.5)	5.0 (4.0, 8.0)	7.0 (4.3, 12.0)	10.0 (6.0, 14.0)	5.5 (3.0, 8.0)
**ER visits**	18 (22.2)	9 (11.3)	15 (18.8)	52 (61.2)	12 (14.8)
**Outpatient visits**	68 (84.0)	51 (63.8)	75 (93.8)	78 (91.8)	73 (90.1)
**Number of EGPA-related visits, per person per year***†					
**Hospitalizations**					
Mean (SD)	0.6 (1.1)	0.2 (0.5)	0.4 (0.9)	0.4 (0.7)	0.7 (2.4)
Median (IQR)	0.0 (0.0, 0.6)	0.0 (0.0, 0.0)	0.0 (0.0, 0.6)	0.0 (0.0, 0.6)	0.0 (0.0, 0.3)
**ER visits**					
Mean (SD)	0.3 (0.6)	0.1 (0.2)	0.3 (1.0)	0.9 (1.5)	0.2 (0.6)
Median (IQR)	0.0 (0.0, 0.0)	0.0 (0.0, 0.0)	0.0 (0.0, 0.0)	0.5 (0.0, 1.0)	0.0 (0.0, 0.0)
**Outpatient visits**					
**Overall outpatient visits**					
Mean (SD)	11.3 (78.7)	5.9 (32.5)	4.7 (5.7)	3.9 (4.4)	3.8 (2.8)
Median (IQR)	2.4 (1.1, 4.1)	2.2 (0.0, 4.1)	3.4 (2.0, 5.5)	3.2 (1.6, 5.0)	3.0 (1.9, 5.2)
**Unscheduled outpatient visits**					
Mean (SD)	0.6 (0.9)	0.6 (1.2)	0.9 (1.0)	0.9 (0.9)	0.4 (0.6)
Median (IQR)	0.4 (0.0, 0.9)	0.0 (0.0, 0.7)	0.6 (0.0, 1.2)	0.7 (0.0, 1.4)	0.0 (0.0, 0.7)

HCRU among patients with EGPA (N=407)	France<br>n=81	Germany<br>n=80	Italy<br>n=80	Spain<br>n=85	UK<br>n=81
**Proportion of patients with any occurrence of tests related to the complications and monitoring of adverse effects of using immunosuppressive medications,*** n (%)					
EGPA-related medical procedure (e.g., plasma exchange)	11 (13.6)	2 (2.5)	7 (8.8)	24 (28.2)	8 (9.9)
Bone mineral density testing	13 (16.0)	16 (20.0)	34 (42.5)	32 (37.6)	23 (28.4)
Cataract removal	2 (2.5)	0 (0.0)	2 (2.5)	1 (1.2)	0 (0.0)
Imaging tests	12 (14.8)	14 (17.5)	24 (30.0)	46 (54.1)	20 (24.7)
**Number of other tests, per person per year*†**					
**EGPA-related medical procedure (e.g., plasma exchange)**					
Mean (SD)	1.2 (1.0)	0.7 (0.7)	1.5 (1.3)	2.2 (3.7)	1.2 (0.9)
Median (IQR)	0.7 (0.5, 1.9)	0.7 (0.2, 1.1)	1.2 (0.6, 1.7)	1.1 (0.5, 2.2)	1.0 (0.7, 1.3)
**Bone mineral density testing**					
Mean (SD)	0.7 (0.4)	0.7 (0.4)	0.8 (0.4)	0.8 (0.6)	0.6 (0.2)
Median (IQR)	0.6 (0.5, 0.9)	0.6 (0.5, 0.8)	0.7 (0.5, 1.1)	0.7 (0.5, 0.9)	0.6 (0.5, 0.7)
**Cataract removal**					
Mean (SD)	1.5 (1.2)	N/A	0.5 (0.4)	0.4 (0.0)	N/A
Median (IQR)	1.5 (0.7, 2.4)	N/A	0.5 (0.2, 0.7)	0.4 (0.4, 0.4)	N/A
**Imaging tests**					
Mean (SD)	1.5 (0.5)	1.0 (0.9)	1.1 (0.5)	1.8 (1.8)	1.3 (1.1)
Median (IQR)	1.2 (1.1, 1.9)	0.7 (0.4, 1.2)	1.2 (0.7, 1.5)	1.3 (0.6, 2.4)	0.9 (0.6, 1.4)

*EGPA-related visits and tests were assessed between index date and EOF. †The number of hospitalizations, ER visits, outpatient visits, and various types of tests were annualized.

CT, computed tomography; EGPA, eosinophilic granulomatosis with polyangiitis; EOF, end of follow-up; ER, emergency room; HCRU, healthcare resource utilization; IQR, interquartile range; MRI, magnetic resonance imaging; N/A, not available; SD, standard deviation; UK, United Kingdom.

Table S8. Pooled analysis of treatment use in patients with HES or EGPA overall and by country.

**Therapies used by treatment category**[*†]	**Overall N=687**	**France n=142**	**Germany n=133**	**Italy n=132**	**Spain n=137**	**UK n=143**
**OCS, n (%)**	**652 (94.9)**	**128 (90.1)**	**133 (100.0)**	**124 (93.9)**	**133 (97.1)**	**134 (93.7)**
Patients with a reported maximum daily dose for maintenance therapy,[‡] n (%)	504 (73.4)	98 (69.0)	87 (65.4)	100 (75.8)	104 (75.9)	115 (80.4)
Maximum daily OCS dose (mg)						
Mean (SD)	30.7 (19.5)	34.2 (19.4)	28.5 (19.1)	21.8 (16.5)	36.9 (20.0)	31.8 (19.2)
Median (IQR)	30.0 (12.0, 50.0)	32.5 (15.0, 60.0)	25.0 (10.0, 50.0)	15.5 (10.0, 30.0)	35.0 (15.0, 60.0)	30.0 (15.0, 50.0)
Duration of OCS use, months						
Mean (SD)	26.8 (28.9)	20.3 (16.9)	22.8 (17.4)	40.1 (44.1)	28.3 (33.9)	24.3 (22.5)
Median (IQR)	20.7 (9.0, 33.8)	17.6 (7.9, 27.0)	21.3 (9.1, 29.4)	27.3 (16.5, 49.1)	18.6 (5.9, 34.1)	20.2 (11.4, 34.0)
**Prednisone or prednisolone, n (%)**	549 (79.9)	111 (78.2)	121 (91.0)	86 (65.2)	114 (83.2)	117 (81.8)
Patients with a reported maximum daily dose for maintenance therapy,[‡] n (%)	426 (62.0)	85 (59.9)	78 (58.6)	71 (53.8)	89 (65.0)	103 (72.0)
Maximum daily dose (mg)						
Mean (SD)	31.5 (19.4)	33.5 (18.4)	29.2 (19.4)	24.9 (17.5)	36.7 (20.2)	31.5 (19.5)
Median (IQR)	30.0 (12.5, 50.0)	30.0 (20.0, 50.0)	25.0 (10.0, 50.0)	25.0 (10.0, 50.0)	30.0 (15.0, 60.0)	35.0 (12.5, 50.0)
Duration, months, Mean (SD)	27.6 (29.7)	19.8 (14.8)	22.7 (16.8)	41.9 (48.2)	30.1 (35.7)	27.2 (23.2)
Duration, months, Median (IQR)	20.6 (11.1, 34.1)	17.6 (7.9, 25.7)	21.1 (9.6, 29.2)	29.1 (14.8, 56.1)	18.7 (8.2, 39.4)	21.8 (13.9, 35.9)
**Methylprednisolone**, n (%)	144 (21.0)	30 (21.1)	11 (8.3)	40 (30.3)	21 (15.3)	42 (29.4)
Patients with a reported maximum daily dose for maintenance therapy,[‡] n (%)	79 (11.5)	15 (10.6)	7 (5.3)	30 (22.7)	13 (9.5)	14 (9.8)
Maximum daily dose (mg)						

Therapies used by treatment category*†	Overall N=687	France n=142	Germany n=133	Italy n=132	Spain n=137	UK n=143
Mean (SD)	24.7 (18.7)	34.1 (23.5)	25.9 (15.4)	13.8 (10.8)	37.8 (19.0)	25.4 (15.8)
Median (IQR)	20.0 (10.0, 40.0)	40.0 (10.0, 60.0)	32.0 (10.0, 32.0)	10.0 (5.0, 16.0)	40.0 (20.0, 50.0)	25.0 (10.0, 30.0)
Duration, months, Mean (SD)	13.0 (21.9)	7.5 (20.7)	16.1 (28.7)	27.6 (29.1)	12.8 (12.8)	5.0 (9.9)
Duration, months, Median (IQR)	2.1 (0.1, 21.6)	0.2 (0.1, 1.6)	2.0 (0.5, 29.7)	22.1 (11.0, 29.5)	7.3 (3.3, 26.8)	0.2 (0.1, 2.9)
**Cortisone**, n (%)	**33 (4.8)**	**9 (6.3)**	**4 (3.0)**	**8 (6.1)**	**4 (2.9)**	**8 (5.6)**
Patients with a reported maximum daily dose for maintenance therapy,‡ n (%)	16 (2.3)	7 (4.9)	2 (1.5)	1 (0.8)	3 (2.2)	3 (2.1)
Maximum daily dose (mg),						
Mean (SD)	30.2 (22.8)	27.9 (24.8)	10.0 (7.1)	12.5 (0.0)	41.7 (23.6)	43.3 (20.8)
Median (IQR)	17.5 (10.0, 55.0)	10.0 (10.0, 60.0)	10.0 (5.0, 15.0)	12.5 (12.5, 12.5)	50.0 (15.0, 60.0)	50.0 (20.0, 60.0)
Duration, months, Mean (SD)	15.1 (12.2)	17.8 (11.6)	19.3 (12.3)	N/A	14.2 (15.4)	9.2 (11.7)
Duration, months, Median (IQR)	15.5 (1.2, 26.2)	19.0 (8.8, 25.6)	18.2 (10.4, 28.1)	N/A	13.6 (0.9, 27.5)	3.5 (0.5, 20.0)
**Immunosuppressive or cytotoxic agents,** n (%)	**384 (55.9)**	**62 (43.7)**	**77 (57.9)**	**81 (61.4)**	**76 (55.5)**	**88 (61.5)**
**Azathioprine,** n (%)	**150 (21.8)**	**22 (15.5)**	**35 (26.3)**	**35 (26.5)**	**21 (15.3)**	**37 (25.9)**
Duration, months, Mean (SD)	25.8 (26.7)	14.1 (9.5)	27.5 (16.8)	28.4 (24.7)	27.1 (23.5)	27.9 (40.0)
Duration, months, Median (IQR)	19.1 (10.0, 31.9)	12.0 (6.2, 21.9)	22.3 (16.3, 34.5)	26.2 (10.2, 35.3)	20.1 (5.3, 43.0)	14.7 (10.1, 24.2)
**Cyclophosphamide,** n (%)	**95 (13.8)**	**9 (6.3)**	**29 (21.8)**	**24 (18.2)**	**15 (10.9)**	**18 (12.6)**
Duration, months						
Mean (SD)	8.2 (8.5)	3.1 (2.0)	8.4 (8.2)	15.5 (12.8)	8.2 (7.4)	5.2 (4.9)
Median (IQR)	4.4 (3.0, 11.0)	2.6 (1.6, 3.2)	5.1 (3.0, 10.5)	12.4 (5.0, 18.9)	4.4 (3.0, 12.2)	3.7 (3.2, 5.3)
**Cyclosporine,** n (%)	**29 (4.2)**	**5 (3.5)**	**4 (3.0)**	**6 (4.5)**	**7 (5.1)**	**7 (4.9)**
Duration, months, Mean (SD)	14.7 (15.2)	13.8 (17.9)	10.5 (4.6)	23.6 (23.1)	5.1 (3.5)	17.4 (15.5)
Duration, months, Median (IQR)	7.9 (5.2, 22.1)	5.8 (4.3, 23.2)	12.8 (5.2, 13.6)	23.2 (3.9, 43.2)	5.9 (2.3, 7.9)	12.2 (5.4, 26.5)

**Therapies used by treatment category**[*†]	**Overall N=687**	**France n=142**	**Germany n=133**	**Italy n=132**	**Spain n=137**	**UK n=143**
**Hydroxyurea,** n (%)	**28 (4.1)**	**6 (4.2)**	**6 (4.5)**	**4 (3.0)**	**6 (4.4)**	**6 (4.2)**
Duration, months, Mean (SD)	16.7 (19.0)	31.6 (31.0)	9.2 (4.9)	24.3 (20.0)	16.1 (16.0)	12.2 (20.8)
Duration, months, Median (IQR)	8.9 (4.8, 26.5)	32.4 (5.0, 58.2)	8.4 (4.9, 12.0)	24.3 (10.2, 38.4)	9.1 (4.9, 35.9)	4.6 (1.0, 8.6)
**Immunoglobulin (intravenous**), n (%)	**23 (3.3)**	**10 (7.0)**	**3 (2.3)**	**5 (3.8)**	**2 (1.5)**	**3 (2.1)**
Duration, months, Mean (SD)	7.5 (13.2)	8.6 (12.7)	2.5 (2.4)	15.5 (22.2)	2.0 (2.4)	2.2 (1.9)
Duration, months, Median (IQR)	2.8 (0.2, 7.5)	0.0 (0.0, 14.8)	2.5 (0.8, 4.2)	6.9 (1.6, 29.4)	2.0 (0.3, 3.6)	2.5 (0.2, 4.0)
**Interferon-alpha,** n (%)	**19 (2.8)**	**3 (2.1)**	**9 (6.8)**	**3 (2.3)**	**2 (1.5)**	**2 (1.4)**
Duration, months, Mean (SD)	10.5 (7.6)	7.7 (9.2)	12.1 (6.5)	1.2 (0.0)	4.0 (0.0)	15.9 (9.9)
Duration, months, Median (IQR)	11.3 (4.0, 12.5)	4.2 (0.8, 18.2)	11.9 (9.9, 12.3)	1.2 (1.2, 1.2)	4.0 (4.0, 4.0)	15.9 (8.9, 22.8)
**Leflunomide,** n (%)	**12 (1.7)**	**2 (1.4)**	**2 (1.5)**	**3 (2.3)**	**4 (2.9)**	**1 (0.7)**
Duration, months, Mean (SD)	6.6 (7.8)	0.0 (0.0)	7.1 (0.0)	0.5 (0.0)	9.7 (9.0)	N/A
Duration, months, Median (IQR)	4.0 (0.5, 7.9)	0.0 (0.0, 0.0)	7.1 (7.1, 7.1)	0.5 (0.5, 0.5)	5.9 (3.9, 15.4)	N/A
**Methotrexate,** n (%)	**106 (15.4)**	**20 (14.1)**	**17 (12.8)**	**23 (17.4)**	**29 (21.2)**	**17 (11.9)**
Duration, months, Mean (SD)	24.4 (28.8)	14.7 (12.5)	18.3 (12.2)	42.0 (52.6)	22.5 (18.8)	22.7 (11.4)
Duration, months, Median (IQR)	16.9 (9.4, 30.4)	12.1 (6.0, 17.5)	16.8 (9.4, 19.5)	25.2 (14.3, 44.6)	16.9 (8.6, 28.7)	24.1 (11.9, 33.6)
**Mycophenolate,** n (%)	**37 (5.4)**	**1 (0.7)**	**1 (0.8)**	**3 (2.3)**	**11 (8.0)**	**21 (14.7)**
Duration, months, Mean (SD)	20.1 (21.6)	66.0 (0.0)	5.8 (0.0)	10.6 (3.1)	11.2 (7.6)	24.0 (24.4)
Duration, months, Median (IQR)	15.7 (7.2, 20.1)	66.0 (66.0, 66.0)	5.8 (5.8, 5.8)	10.3 (7.7, 13.8)	9.2 (4.6, 18.3)	18.2 (11.0, 30.4)
**Biologics,** n (%)	**295 (42.9)**	**72 (50.7)**	**34 (25.6)**	**79 (59.8)**	**62 (45.3)**	**48 (33.6)**
**Mepolizumab,** n (%)	**117 (17.0)**	**27 (19.0)**	**9 (6.8)**	**47 (35.6)**	**21 (15.3)**	**13 (9.1)**
Duration, months, Mean (SD)	10.9 (10.1)	10.7 (10.3)	13.1 (15.5)	10.9 (8.6)	11.9 (12.0)	8.1 (7.4)
Duration, months, Median (IQR)	8.4 (3.4, 15.4)	7.3 (4.8, 11.7)	5.7 (1.6, 22.7)	9.9 (3.0, 17.7)	10.4 (3.5, 13.8)	3.9 (2.8, 14.7)
**Benralizumab,** n (%)	**60 (8.7)**	**12 (8.5)**	**12 (9.0)**	**13 (9.8)**	**12 (8.8)**	**11 (7.7)**
Duration, months, Mean (SD)	9.5 (7.9)	8.9 (10.0)	13.6 (9.3)	11.2 (6.0)	6.5 (4.1)	4.4 (5.7)
Duration, months, Median (IQR)	8.1 (3.0, 14.2)	7.7 (3.0, 8.9)	12.1 (6.9, 16.2)	14.3 (6.7, 15.1)	5.6 (4.8, 9.4)	2.3 (0.9, 3.9)

Therapies used by treatment category*†	Overall N=687	France n=142	Germany n=133	Italy n=132	Spain n=137	UK n=143
**Dupilumab,** n (%)	**23 (3.3)**	**5 (3.5)**	**5 (3.8)**	**7 (5.3)**	**0 (0.0)**	**6 (4.2)**
Duration, months, Mean (SD)	4.2 (3.5)	4.2 (5.5)	2.8 (1.5)	5.3 (3.8)	N/A	N/A
Duration, months, Median (IQR)	3.8 (1.6, 4.9)	1.6 (0.4, 10.5)	3.5 (2.5, 3.8)	4.7 (4.0, 5.5)	N/A	N/A
**Omalizumab,** n (%)	**31 (4.5)**	**2 (1.4)**	**6 (4.5)**	**9 (6.8)**	**11 (8.0)**	**3 (2.1)**
Duration, months, Mean (SD)	12.0 (10.5)	7.3 (10.4)	13.6 (10.7)	6.5 (5.9)	14.0 (12.2)	18.8 (12.9))
Duration, months, Median (IQR)	7.3 (3.9, 18.9)	7.3 (0.0, 14.7)	10.3 (6.0, 17.2)	5.0 (3.8, 7.3)	13.5 (3.4, 26.7)	21.9 (4.6, 29.9)
**Reslizumab,** n (%)	**29 (4.2)**	**11 (7.7)**	**1 (0.8)**	**3 (2.3)**	**10 (7.3)**	**4 (2.8)**
Duration, months, Mean (SD)	3.9 (3.1)	3.8 (3.6)	6.2 (0.0)	0.0 (0.0)	4.2 (3.1)	4.1 (2.6)
Duration, months, Median (IQR)	3.3 (1.6, 5.5)	2.9 (1.6, 5.5)	6.2 (6.2, 6.2)	0.0 (0.0, 0.0)	3.9 (2.5, 5.1)	4.1 (2.1, 6.0)
**Rituximab,** n (%)	**108 (15.7)**	**37 (26.1)**	**13 (9.8)**	**15 (11.4)**	**24 (17.5)**	**19 (13.3)**
Duration, months, Mean (SD)	14.0 (16.0)	14.6 (14.5)	10.0 (7.6)	15.3 (10.5)	18.4 (25.0)	8.2 (9.3)
Duration, months, Median (IQR)	9.5 (3.6, 20.5)	7.7 (3.5, 23.6)	9.4 (2.8, 18.2)	12.6 (5.0, 22.2)	12.3 (4.3, 23.2)	5.5 (0.7, 12.0)
**Other treatments used related to the complications and adverse effects of immunosuppressive medications,§ n (%)**	**369 (53.7)**	**62 (43.7)**	**72 (54.1)**	**83 (62.9)**	**73 (53.3)**	**79 (55.2)**
Treatments for the improvement of bone mineral density¶	246 (35.8)	39 (27.5)	45 (33.8)	58 (43.9)	52 (38.0)	52 (36.4)
Treatment for infections in relation to HES/EGPA therapies**	102 (14.8)	21 (14.8)	16 (12.0)	21 (15.9)	22 (16.1)	22 (15.4)
Thyroid hormone replacement treatments	45 (6.6)	5 (3.5)	14 (10.5)	5 (3.8)	10 (7.3)	11 (7.7)
Insulin	38 (5.5)	6 (4.2)	8 (6.0)	6 (4.5)	5 (3.6)	13 (9.1)
Folic acid supplements	148 (21.5)	22 (15.5)	25 (18.8)	31 (23.5)	39 (28.5)	31 (21.7)

Therapies used by treatment category*†	Overall N=687	France n=142	Germany n=133	Italy n=132	Spain n=137	UK n=143
**Ongoing treatments received at EOF among pooled patients with HES and EGPA,[††] n (%)**						
**OCS**	**379 (55.2)**	**71 (50.0)**	**79 (59.4)**	**79 (59.8)**	**71 (51.8)**	**79 (55.2)**
Prednisone or prednisolone	322 (46.9)	63 (44.4)	73 (54.9)	56 (42.4)	59 (43.1)	71 (49.7)
Methylprednisolone	42 (6.1)	3 (2.1)	3 (2.3)	20 (15.2)	10 (7.3)	6 (4.2)
Cortisone	16 (2.3)	5 (3.5)	3 (2.3)	4 (3.0)	2 (1.5)	2 (1.4)
**Immunosuppressive and cytotoxic agents,** n (%)	**223 (32.5)**	**34 (23.9)**	**51 (38.3)**	**41 (31.1)**	**40 (29.2)**	**57 (39.9)**
Azathioprine	88 (12.8)	14 (9.9)	25 (18.8)	16 (12.1)	9 (6.6)	24 (16.8)
Cyclophosphamide	22 (3.2)	2 (1.4)	8 (6.0)	3 (2.3)	7 (5.1)	2 (1.4)
Cyclosporine	13 (1.9)	3 (2.1)	1 (0.8)	3 (2.3)	3 (2.2)	3 (2.1)
Hydroxyurea	9 (1.3)	3 (2.1)	2 (1.5)	2 (1.5)	0 (0.0)	2 (1.4)
Immunoglobulin (intravenous)	3 (0.4)	1 (0.7)	0 (0.0)	2 (1.5)	0 (0.0)	0 (0.0)
Interferon-alpha	7 (1.0)	1 (0.7)	2 (1.5)	2 (1.5)	0 (0.0)	2 (1.4)
Leflunomide	4 (0.6)	0 (0.0)	1 (0.8)	1 (0.8)	2 (1.5)	0 (0.0)
Methotrexate	60 (8.7)	9 (6.3)	12 (9.0)	11 (8.3)	17 (12.4)	11 (7.7)
Mycophenolate	25 (3.6)	1 (0.7)	1 (0.8)	2 (1.5)	5 (3.6)	16 (11.2)
**Biologics,** n (%)	**215 (31.3)**	**51 (35.9)**	**25 (18.8)**	**54 (40.9)**	**50 (36.5)**	**35 (24.5)**
Mepolizumab	87 (12.7)	19 (13.4)	6 (4.5)	33 (25.0)	18 (13.1)	11 (7.7)
Benralizumab	49 (7.1)	9 (6.3)	10 (7.5)	11 (8.3)	11 (8.0)	8 (5.6)
Dupilumab	15 (2.2)	4 (2.8)	3 (2.3)	2 (1.5)	0 (0.0)	6 (4.2)
Omalizumab	21 (3.1)	0 (0.0)	6 (4.5)	3 (2.3)	9 (6.6)	3 (2.1)
Reslizumab	21 (3.1)	8 (5.6)	1 (0.8)	2 (1.5)	8 (5.8)	2 (1.4)
Rituximab	55 (8.0)	19 (13.4)	6 (4.5)	6 (4.5)	13 (9.5)	11 (7.7)

*Includes 407 patients with EGPA and 280 patients with HES. Treatment patterns were assessed between diagnosis and EOF. Receipt of one or multiple OCS drugs was counted as a single therapy. †For patients who received ≥1 prescriptions for a drug, the duration of therapy was calculated as the sum of durations of therapy across all prescriptions. For patients who received ≥1 prescriptions for a drug, the maximum dosage was reported as the maximum dosage across all prescriptions. ‡Patients with reported maximum daily dose for maintenance therapy over 60 mg were removed from the summary of maximum daily dose statistics, as these values seemed most likely to reflect dosing for burst treatment episodes instead of for maintenance therapy. §Treatment patterns for other treatments were assessed between index date and EOF. ¶ Examples of treatments for the improvement of bone mineral density include alendronate, risedronate sodium, ibandronate, and zoledronic acid. **Examples of treatments for infection in relation to HES/EGPA therapies include antitubercular agents, acyclovir, valacyclovir, and fluconazole. ††Includes 407 patients with EGPA and 280 patients with HES. Ongoing treatments are treatments that the patient was indicated as taking at EOF.

EGPA, eosinophilic granulomatosis with polyangiitis; EOF, end of follow-up; HES, hypereosinophilic syndrome; IQR, interquartile range; N/A, not available; OCS, oral corticosteroid; SD, standard deviation; UK, United Kingdom.

Table S9. Pooled comorbidities data among patients with HES and EGPA (overall and by country).

Clinical characteristics,* n (%)	Overall† N=687	France n=142	Germany n=133	Italy n=132	Spain n=137	UK n=143
**Comorbidities**						
Asthma	425 (61.9)	80 (56.3)	75 (56.4)	91 (68.9)	89 (65.0)	90 (62.9)
Hypertension	254 (37.0)	50 (35.2)	53 (39.8)	62 (47.0)	44 (32.1)	45 (31.5)
Vasculitis	244 (35.5)	46 (32.4)	46 (34.6)	56 (42.4)	46 (33.6)	50 (35.0)
Anxiety or depression	242 (35.2)	60 (42.3)	28 (21.1)	64 (48.5)	34 (24.8)	56 (39.2)
Lower respiratory disease, other than asthma and COPD	116 (16.9)	31 (21.8)	14 (10.5)	23 (17.4)	25 (18.2)	23 (16.1)
Obesity	112 (16.3)	14 (9.9)	27 (20.3)	18 (13.6)	30 (21.9)	23 (16.1)
Osteoporosis	103 (15.0)	12 (8.5)	14 (10.5)	31 (23.5)	27 (19.7)	19 (13.3)
Glomerulonephritis	90 (13.1)	21 (14.8)	21 (15.8)	19 (14.4)	11 (8.0)	18 (12.6)
Diabetes	70 (10.2)	8 (5.6)	14 (10.5)	10 (7.6)	16 (11.7)	22 (15.4)
Liver disease	35 (5.1)	7 (4.9)	8 (6.0)	4 (3.0)	10 (7.3)	6 (4.2)
Rheumatoid arthritis	28 (4.1)	11 (7.7)	7 (5.3)	4 (3.0)	4 (2.9)	2 (1.4)
Other‡	30 (4.4)	5 (3.5)	1 (0.8)	16 (12.1)	6 (4.4)	2 (1.4)
**Cancer§**	28 (4.1)	6 (4.2)	1 (0.8)	5 (3.8)	7 (5.1)	9 (6.3)
Leukemia	8 (1.2)	1 (0.7)	1 (0.8)	0 (0.0)	4 (2.9)	2 (1.4)
Lung	6 (0.9)	2 (1.4)	0 (0.0)	1 (0.8)	1 (0.7)	2 (1.4)
Prostate	4 (0.6)	1 (0.7)	0 (0.0)	2 (1.5)	0 (0.0)	1 (0.7)
Breast	2 (0.3)	1 (0.7)	0 (0.0)	0 (0.0)	0 (0.0)	1 (0.7)
Skin	2 (0.3)	1 (0.7)	0 (0.0)	0 (0.0)	1 (0.7)	0 (0.0)
Colon	1 (0.1)	0 (0.0)	0 (0.0)	0 (0.0)	1 (0.7)	0 (0.0)
Rectum	1 (0.1)	0 (0.0)	0 (0.0)	1 (0.8)	0 (0.0)	0 (0.0)

Clinical characteristics,* n (%)	Overall† N=687	France n=142	Germany n=133	Italy n=132	Spain n=137	UK n=143
Cavum	1 (0.1)	0 (0.0)	0 (0.0)	0 (0.0)	0 (0.0)	1 (0.7)
Anus	1 (0.1)	0 (0.0)	0 (0.0)	0 (0.0)	0 (0.0)	1 (0.7)
Renal	1 (0.1)	0 (0.0)	0 (0.0)	0 (0.0)	0 (0.0)	1 (0.7)
Squamous cell carcinoma	1 (0.1)	0 (0.0)	0 (0.0)	1 (0.8)	0 (0.0)	0 (0.0)
Basal cell carcinoma	1 (0.1)	1 (0.7)	0 (0.0)	0 (0.0)	0 (0.0)	0 (0.0)

*Clinical characteristics were assessed between EGPA/HES diagnosis and EOF (i.e., last physician encounter or death). †Includes 407 patients with EGPA and 280 patients with HES ‡Other reported comorbidities included hay fever (4), sinusitis (3), osteoporosis (2), rhinitis (2), allergy (1), angioedema (1), atrial fibrillation (1), bowel infarction (1), cardiac ischemia (1), colitis (1), coronary artery disease (1), esophagitis (1), eosinophilic gastroenteritis (1), fatty liver disease (1), heart failure (1), hives (1), hypothyroidism (1), multiple sclerosis (1), obstructive sleep apnea symptoms (1), oligoarthritis (1), peripheral neuropathy (1), psoriasis (1), restriction (1), skin ulcer (1), and strabismus (1). §Patients could be indicated for multiple cancers. The categories are not mutually exclusive.

COPD, chronic obstructive pulmonary disease; EGPA, eosinophilic granulomatosis with polyangiitis; EOF, end of follow-up; HES, hypereosinophilic syndrome; UK, United Kingdom.

Table S10. Pooled clinical manifestations and symptom severity in HES and EGPA, overall and by country.

Clinical manifestations by organ involvement, n (%)*	Overall N=687	France n=142	Germany n=133	Italy n=132	Spain n=137	UK n=143
**Constitutional**	**343 (49.9)**	**79 (55.6)**	**62 (46.6)**	**57 (43.2)**	**74 (54.0)**	**71 (49.7)**
Fatigue	313 (45.6)	75 (52.8)	54 (40.6)	54 (40.9)	67 (48.9)	63 (44.1)
Proportion of manifestations classified as moderate to severe†	240 (76.6)	65 (86.7)	32 (59.3)	39 (72.3)	50 (74.7)	54 (85.7)
Pain‡	79 (11.5)	18 (12.7)	12 (9.0)	13 (9.8)	24 (17.5)	12 (8.4)
Proportion of manifestations classified as moderate to severe†	63 (79.7)	15 (83.3)	9 (75.0)	11 (84.6)	23 (95.8)	5 (41.7)
**Lung**	**314 (45.7)**	**67 (47.2)**	**56 (42.1)**	**63 (47.7)**	**74 (54.0)**	**54 (37.8)**
Shortness of breath	203 (29.5)	36 (25.4)	37 (27.8)	42 (31.8)	47 (34.3)	41 (28.7)
Proportion of manifestations classified as moderate to severe†	150 (73.9)	27 (75.0)	27 (73.0)	30 (71.4)	39 (83.0)	27 (65.8)
Asthma§	167 (24.3)	36 (25.4)	26 (19.5)	44 (33.3)	46 (33.6)	15 (10.5)
Proportion of manifestations classified as moderate to severe†	138 (82.6)	31 (86.1)	17 (65.4)	37 (84.1)	44 (95.6)	9 (60.0)
Pulmonary infiltration¶	35 (5.1)	16 (11.3)	4 (3.0)	5 (3.8)	4 (2.9)	6 (4.2)
Proportion of manifestations classified as moderate to severe†	27 (77.2)	13 (81.3)	3 (75.0)	3 (60.0)	3 (75.0)	5 (83.3)
**ENT**	**256 (37.3)**	**45 (31.7)**	**43 (32.3)**	**50 (37.9)**	**66 (48.2)**	**52 (36.4)**
Rhinitis/Pharyngitis**	209 (30.4)	39 (27.5)	32 (24.1)	42 (31.8)	55 (40.1)	41 (28.7)
Sinusitis††	90 (13.1)	13 (9.2)	19 (14.3)	18 (13.6)	21 (15.3)	19 (13.3)
Proportion of manifestations classified as moderate to severe†	63 (70.0)	11 (84.7)	12 (63.2)	13 (72.3)	15 (71.4)	12 (63.2)
Ear symptoms‡‡	11 (1.6)	2 (1.4)	1 (0.8)	2 (1.5)	1 (0.7)	5 (3.5)
Proportion of manifestations classified as moderate to severe†	7 (63.6)	1 (50.0)	1 (100.0)	0 (0.0)	0 (0.0)	5 (100.0)
**Skin**	**234 (34.1)**	**37 (26.1)**	**44 (33.1)**	**52 (39.4)**	**54 (39.4)**	**47 (32.9)**
Itch	173 (25.2)	28 (19.7)	30 (22.6)	38 (28.8)	42 (30.7)	35 (24.5)

**Clinical manifestations by organ involvement, n (%)***	**Overall N=687**	**France n=142**	**Germany n=133**	**Italy n=132**	**Spain n=137**	**UK n=143**
Proportion of manifestations classified as moderate to severe†	113 (65.3)	17 (60.7)	23 (76.7)	23 (60.5)	30 (71.4)	20 (57.1)
Hives/urticaria	106 (15.4)	17 (12.0)	22 (16.5)	24 (18.2)	23 (16.8)	20 (14.0)
Proportion of manifestations classified as moderate to severe†	74 (69.9)	11 (64.7)	13 (59.0)	18 (75.0)	16 (69.5)	16 (80.0)
**Gastrointestinal**	**144 (21.0)**	**26 (18.3)**	**34 (25.6)**	**27 (20.5)**	**31 (22.6)**	**26 (18.2)**
Abdominal pain	75 (10.9)	15 (10.6)	18 (13.5)	9 (6.8)	17 (12.4)	16 (11.2)
Proportion of manifestations classified as moderate to severe†	42 (56.0)	7 (46.7)	12 (66.7)	6 (66.7)	8 (47.1)	9 (56.3)
Diarrhea	67 (9.8)	10 (7.0)	11 (8.3)	14 (10.6)	17 (12.4)	15 (10.5)
Proportion of manifestations classified as moderate to severe†	34 (50.7)	4 (40.0)	6 (54.6)	10 (71.4)	7 (41.2)	7 (46.7)
Nausea/vomiting	53 (7.7)	9 (6.3)	14 (10.5)	12 (9.1)	9 (6.6)	9 (6.3)
Proportion of manifestations classified as moderate to severe†	27 (51.0)	4 (44.4)	8 (57.1)	4 (33.3)	5 (55.6)	6 (66.6)
Other gastrointestinal symptoms§§	12 (1.7)	2 (1.4)	1 (0.8)	4 (3.0)	2 (1.5)	3 (2.1)
Proportion of manifestations classified as moderate to severe†	5 (41.7)	0 (0.0)	0 (0.0)	3 (75.0)	1 (50.0)	1 (33.3)
**Neuropsychiatric**	**108 (15.7)**	**17 (12.0)**	**22 (16.5)**	**23 (17.4)**	**21 (15.3)**	**25 (17.5)**
Neuropathies¶¶	104 (15.1)	16 (11.3)	21 (15.8)	23 (17.4)	20 (14.6)	24 (16.8)
Cognitive and mental status change***	8 (1.2)	2 (1.4)	1 (0.8)	1 (0.8)	2 (1.5)	2 (1.4)
Proportion of manifestations classified as moderate to severe†	1 (12.5)	0 (0.0)	0 (0.0)	0 (0.0)	1 (50.0)	0 (0.0)
**Cardiovascular**	**82 (11.9)**	**16 (11.3)**	**13 (9.8)**	**21 (15.9)**	**21 (15.3)**	**11 (7.7)**
Cardiomyopathy	29 (4.2)	8 (5.6)	4 (3.0)	5 (3.8)	8 (5.8)	4 (2.8)
Proportion of manifestations classified as moderate to severe†	14 (48.2)	5 (62.5)	1 (25.0)	1 (20.0)	6 (75.0)	1 (25.0)
Heart failure	21 (3.1)	5 (3.5)	6 (4.5)	3 (2.3)	3 (2.2)	4 (2.8)
Proportion with functional capacity classification†††						
I	14 (66.7)	3 (60.0)	5 (83.3)	3 (100.0)	2 (66.7)	1 (25.0)
II	4 (19.0)	2 (40.0)	0 (0.0)	0 (0.0)	1 (33.3)	1 (25.0)

Clinical manifestations by organ involvement, n (%)*	Overall N=687	France n=142	Germany n=133	Italy n=132	Spain n=137	UK n=143
III	2 (9.5)	0 (0.0)	1 (16.7)	0 (0.0)	0 (0.0)	1 (25.0)
Proportion with objective assessment classification†††						
A	15 (71.4)	4 (80.0)	5 (83.3)	3 (100.0)	2 (66.7)	1 (25.0)
B	3 (14.3)	1 (20.0)	0 (0.0)	0 (0.0)	1 (33.3)	1 (25.0)
C	2 (9.5)	0 (0.0)	1 (16.7)	0 (0.0)	0 (0.0)	1 (25.0)
Ischemic heart disease	21 (3.1)	2 (1.4)	3 (2.3)	9 (6.8)	3 (2.2)	4 (2.8)
Proportion of manifestations classified as moderate to severe†	13 (61.9)	0 (0.0)	2 (66.7)	8 (88.9)	1 (33.3)	2 (50.0)
Valvular disease	13 (1.9)	3 (2.1)	1 (0.8)	1 (0.8)	5 (3.6)	3 (2.1)
Proportion of manifestations classified as moderate to severe†	5 (38.5)	2 (66.6)	0 (0.0)	0 (0.0)	2 (40.0)	1 (33.3)
Other cardiovascular disease‡‡‡	39 (5.7)	7 (4.9)	6 (4.5)	10 (7.6)	10 (7.3)	6 (4.2)
**Renal**	**80 (11.6)**	**17 (12.0)**	**13 (9.8)**	**19 (14.4)**	**11 (8.0)**	**20 (14.0)**
Renal manifestations§§§	80 (11.6)	17 (12.0)	13 (9.8)	19 (14.4)	11 (8.0)	20 (14.0)
**Biopsy confirmed eosinophilic vasculitis or eosinophilic inflammation**	**54 (7.9)**	**19 (13.4)**	**3 (2.3)**	**9 (6.8)**	**13 (9.5)**	**10 (7.0)**
Proportion of manifestations classified as moderate to severe†	38 (70.4)	11 (57.9)	1 (33.3)	7 (77.8)	9 (69.3)	10 (100.0)

*Includes 407 patients with EGPA and 280 patients with HES. The manifestations between index date to EOF are reported. †Severity was documented at first occurrence of a manifestation on the following scale: mild (present but minimal impact), moderate (significant impact on daily activities), severe (incapacitating). Moderate and severe severity have been combined (mild not shown). ‡Includes myalgia/arthralgia manifestation in patients with EGPA and pain manifestation in patients with HES. §Includes severe asthma manifestation in patients with EGPA and asthma manifestation in patients with HES. ¶Includes lung infiltrates manifestation in patients with EGPA and pulmonary infiltration manifestation in patients with HES. **Includes allergic rhinitis manifestation in patients with EGPA and nasal congestion, postnasal drip, and purulent rhinorrhea manifestations in patients with HES. ††Includes paranasal sinusitis manifestation in patients with EGPA and sinus headache/facial pain/pressure manifestation in patients with HES. ‡‡ Includes otitis media manifestation in patients with EGPA and ear-fullness manifestation in patients with HES. §§Includes gastrointestinal bleeding manifestation in patients with EGPA and difficulty in swallowing food manifestation in patients with HES. ¶¶Includes peripheral neuropathy and mononeuritis manifestations in patients with EGPA and sensory neuropathy and motor neuropathy manifestations in patients with HES. ***Includes psychosis manifestation in patients with EGPA and cognitive and mental status change manifestation in patients with HES. †††Severity of heart failure was defined by functional capacity (Class I to IV; where I is no limitation on physical activity and IV is symptoms of heart failure at rest and increasing discomfort with physical activity) and objective assessment (Class A to D; where A is no evidence of

cardiovascular disease and D is severe cardiovascular disease) based on NYHA heart failure criteria. Severity classification was unknown for 1 case from Italy. [‡‡‡]Includes cardiac arrhythmia, peripheral vascular disease, and pericarditis manifestations in patients with EGPA and thromboembolism and arterial hypertension manifestations in patients with HES. Arterial hypertension was not listed in the case report form, but was reported as free-text response by one or more physician respondents. [§§§]Includes glomerulonephritis, proteinuria, and hematuria manifestations in patients with EGPA and kidney failure manifestation in patients with HES.

EGPA, eosinophilic granulomatosis with polyangiitis; ENT, ear, nose, and throat; EOF, end of follow-up; HES, hypereosinophilic syndrome; IQR, interquartile range; NYHA, New York Heart Association; SD, standard deviation; UK, United Kingdom.

Table S11. Pooled HCRU for HES and EGPA, overall and by country.

	**Overall N=687**	**France n=142**	**Germany n=133**	**Italy n=132**	**Spain n=137**	**UK n=143**
**Disease-related visits**						
**Hospitalizations, n (%)**	**234 (34.1)**	**52 (36.6)**	**34 (25.6)**	**50 (37.9)**	**45 (32.8)**	**53 (37.1)**
Average length of stay per hospitalization, days†						
Mean (SD)	9.4 (7.8)	8.7 (8.1)	8.9 (9.3)	9.8 (6.8)	11.6 (6.1)	8.1 (8.4)
Median (IQR)	7.0 (5.0, 12.0)	7.0 (4.0, 10.0)	5.5 (4.0, 8.0)	9.5 (5.0, 12.0)	12.0 (7.9, 14.0)	6.0 (3.0, 10.0)
**ER visits, n (%)**	**178 (25.9)**	**36 (25.4)**	**20 (15.0)**	**22 (16.7)**	**65 (47.4)**	**35 (24.5)**
**Hospitalizations OR ER visits, n (%)**	**303 (44.1)**	**63 (44.4)**	**48 (36.1)**	**58 (43.9)**	**72 (52.6)**	**62 (43.4)**
**Outpatient visits, n (%)**	**588 (85.6)**	**125 (88.0)**	**94 (70.7)**	**116 (87.9)**	**125 (91.2)**	**128 (89.5)**
**Number of disease-related visits, PPPY**						
**Hospitalizations**						
Mean (SD)	0.4 (1.3)	0.5 (1.2)	0.4 (1.3)	0.4 (0.8)	0.3 (0.6)	0.6 (1.9)
Median (IQR)	0.0 (0.0, 0.5)	0.0 (0.0, 0.5)	0.0 (0.0, 0.2)	0.0 (0.0, 0.5)	0.0 (0.0, 0.3)	0.0 (0.0, 0.5)
**ER visits**						
Mean (SD)	0.3 (0.9)	0.3 (0.9)	0.1 (0.4)	0.3 (0.8)	0.6 (1.3)	0.3 (0.7)
Median (IQR)	0.0 (0.0, 0.2)	0.0 (0.0, 0.2)	0.0 (0.0, 0.0)	0.0 (0.0, 0.0)	0.0 (0.0, 0.8)	0.0 (0.0, 0.0)
**Hospitalizations OR ER visits, n (%)**	**303 (44.1)**	**63 (44.4)**	**48 (36.1)**	**58 (43.9)**	**72 (52.6)**	**62 (43.4)**
Mean (SD)	0.8 (1.8)	0.9 (2.0)	0.5 (1.3)	0.6 (1.5)	0.9 (1.7)	0.9 (2.1)
Median (IQR)	0.0 (0.0, 0.9)	0.0 (0.0, 1.0)	0.0 (0.0, 0.6)	0.0 (0.0, 0.9)	0.2 (0.0, 1.2)	0.0 (0.0, 0.9)
**Outpatient visits**						
**Overall outpatient visits**						
Mean (SD)	5.2 (29.4)	8.1 (59.5)	5.8 (25.7)	4.1 (4.9)	4.4 (4.6)	3.8 (3.1)
Median (IQR)	3.0 (1.3, 5.2)	2.5 (1.2, 4.7)	2.6 (0.0, 4.8)	3.2 (1.6, 5.4)	3.6 (1.8, 5.7)	2.9 (1.5, 5.4)
**Unscheduled outpatient visits**						

	Overall N=687	France n=142	Germany n=133	Italy n=132	Spain n=137	UK n=143
Mean (SD)	0.8 (2.2)	0.6 (0.8)	1.1 (4.6)	0.8 (0.9)	0.9 (1.0)	0.7 (1.1)
Median (IQR)	0.5 (0.0, 1.1)	0.4 (0.0, 0.9)	0.3 (0.0, 1.0)	0.6 (0.0, 1.1)	0.7 (0.0, 1.5)	0.3 (0.0, 1.0)
**Proportion of patients with any occurrence of tests related to the complications and monitoring of adverse effects of using immunosuppressive medications, n (%)**						
Imaging tests	253 (36.8)	41 (28.9)	33 (24.8)	53 (40.2)	75 (54.7)	51 (35.7)
Bone mineral density testing	233 (33.9)	37 (26.1)	43 (32.3)	57 (43.2)	49 (35.8)	47 (32.9)
Cataract removal	22 (3.2)	6 (4.2)	0 (0.0)	7 (5.3)	4 (2.9)	5 (3.5)
**Number of other tests, PPPY**						
**Imaging tests**						
Mean (SD)	1.3 (3.5)	2.3 (7.4)	0.7 (1.0)	0.9 (1.0)	1.4 (1.6)	1.3 (2.0)
Median (IQR)	0.7 (0.0, 1.8)	0.8 (0.0, 1.9)	0.0 (0.0, 1.3)	0.7 (0.0, 1.5)	1.1 (0.2, 2.1)	0.7 (0.0, 1.8)
**Bone mineral density testing**						
Mean (SD)	0.4 (0.5)	0.4 (0.5)	0.5 (0.5)	0.6 (0.7)	0.4 (0.5)	0.4 (0.5)
Median (IQR)	0.4 (0.0, 0.7)	0.1 (0.0, 0.6)	0.5 (0.0, 0.7)	0.5 (0.0, 0.9)	0.3 (0.0, 0.7)	0.3 (0.0, 0.7)
**Cataract removal**						
Mean (SD)	0.0 (0.2)	0.1 (0.3)	0.0 (0.0)	0.1 (0.3)	0.0 (0.2)	0.0 (0.1)
Median (IQR)	0.0 (0.0, 0.0)	0.0 (0.0, 0.0)	0.0 (0.0, 0.0)	0.0 (0.0, 0.0)	0.0 (0.0, 0.0)	0.0 (0.0, 0.0)

*Includes patients with 407 EGPA and 280 patients with HES. EGPA- and HES-related visits and tests were assessed between index date and EOF (i.e., last physician encounter or death). [†]Length of stay is calculated among patients with inpatient visits.

EGPA, eosinophilic granulomatosis with polyangiitis; EOF, end of follow-up; ER, emergency room; HCRU, healthcare resource use; HES, hypereosinophilic syndrome; IQR, interquartile range; PPPY, per person per year; SD, standard deviation; UK, United Kingdom.

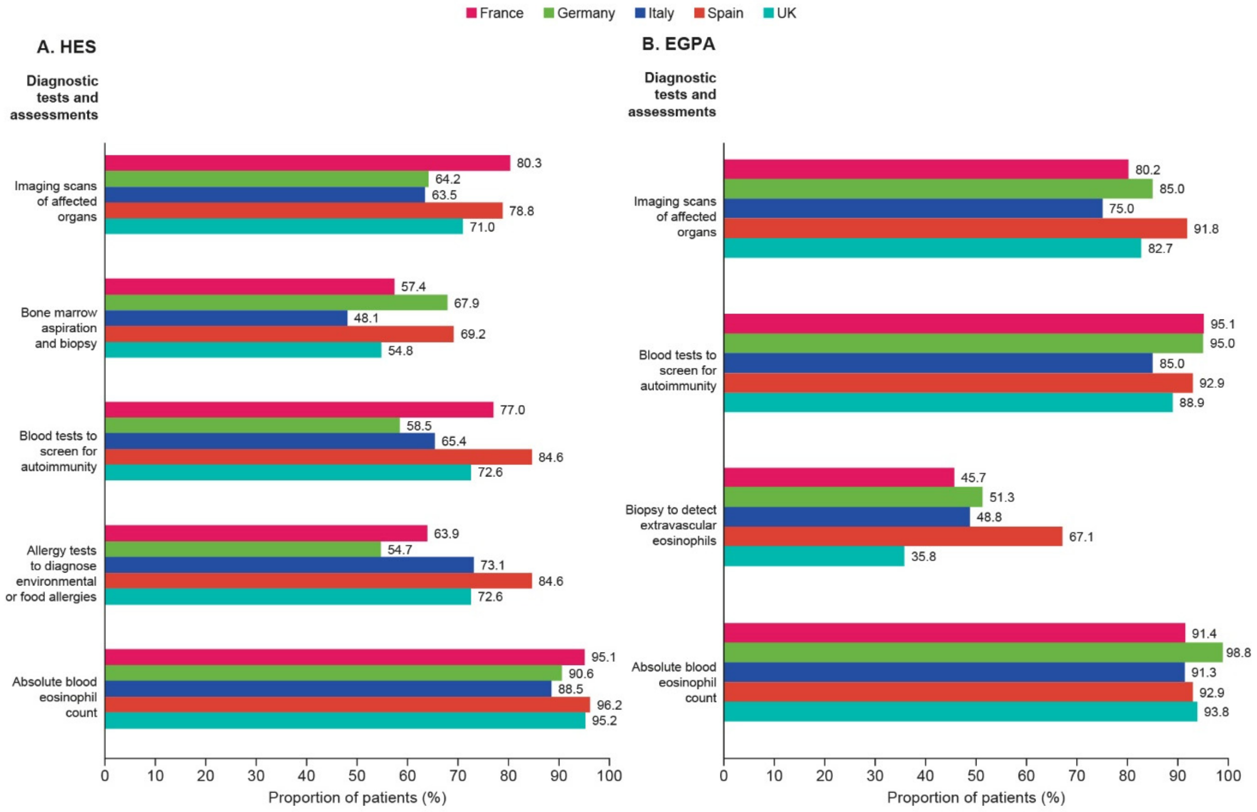

Figure S1. Use of select diagnostic tests and assessments* in A. patients with HES and B. patients with EGPA.

*Diagnostic tests and assessments data for the overall HES and overall EGPA populations have been previously published [17,21]. Categories of diagnostic assessment are not mutually exclusive. Patients may have used multiple diagnostic assessments.

EGPA, eosinophilic granulomatosis with polyangiitis; HES, hypereosinophilic syndrome; UK, United Kingdom.

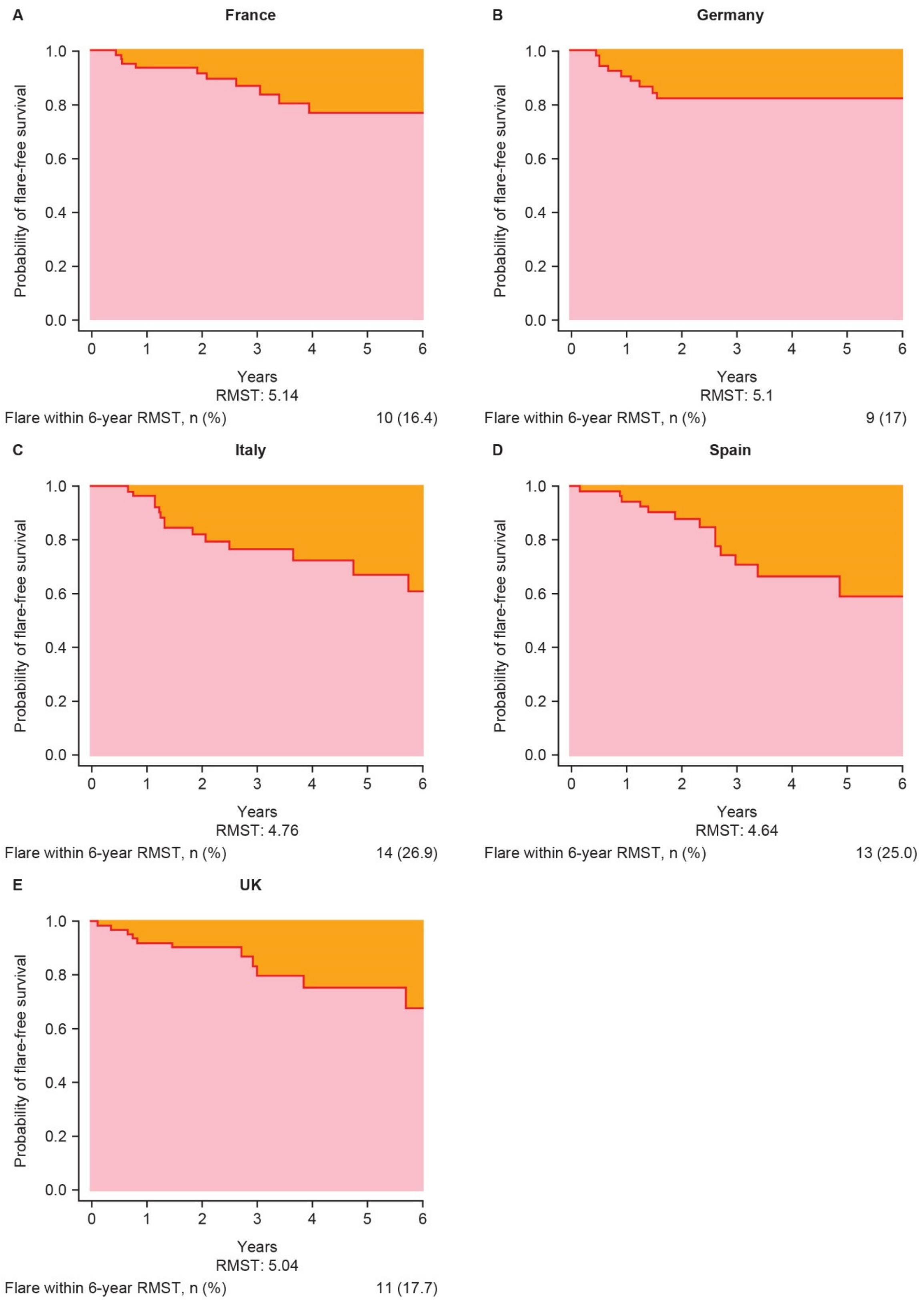

Figure S2. Flare-free survival in patients with HES by country (A-E). HES, hypereosinophilic syndrome; RMST, restricted mean survival time; UK, United Kingdom

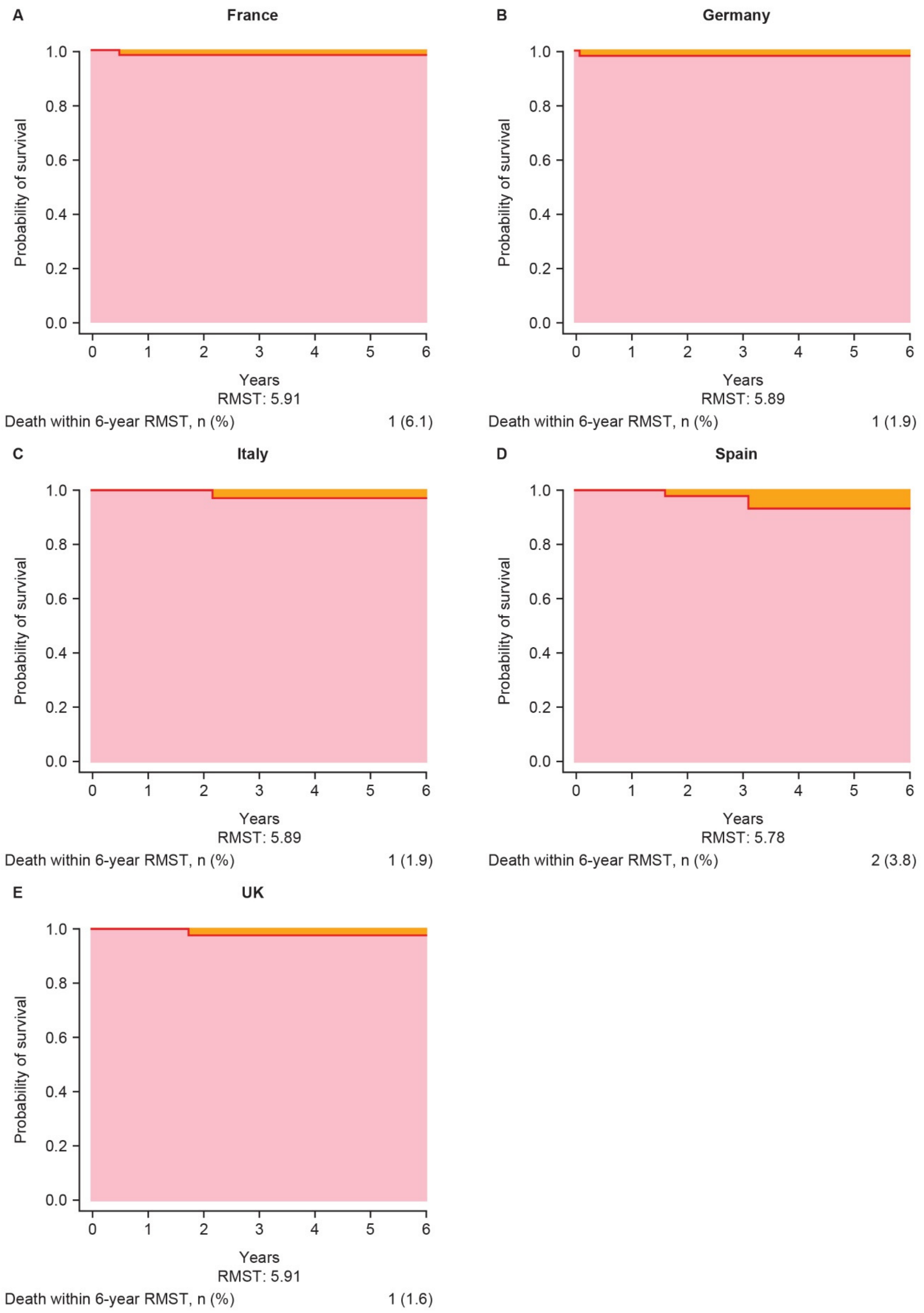

Figure S3. Overall survival in patients with HES by country (A-E). HES, hypereosinophilic syndrome; RMST, restricted mean survival time; UK, United Kingdom.

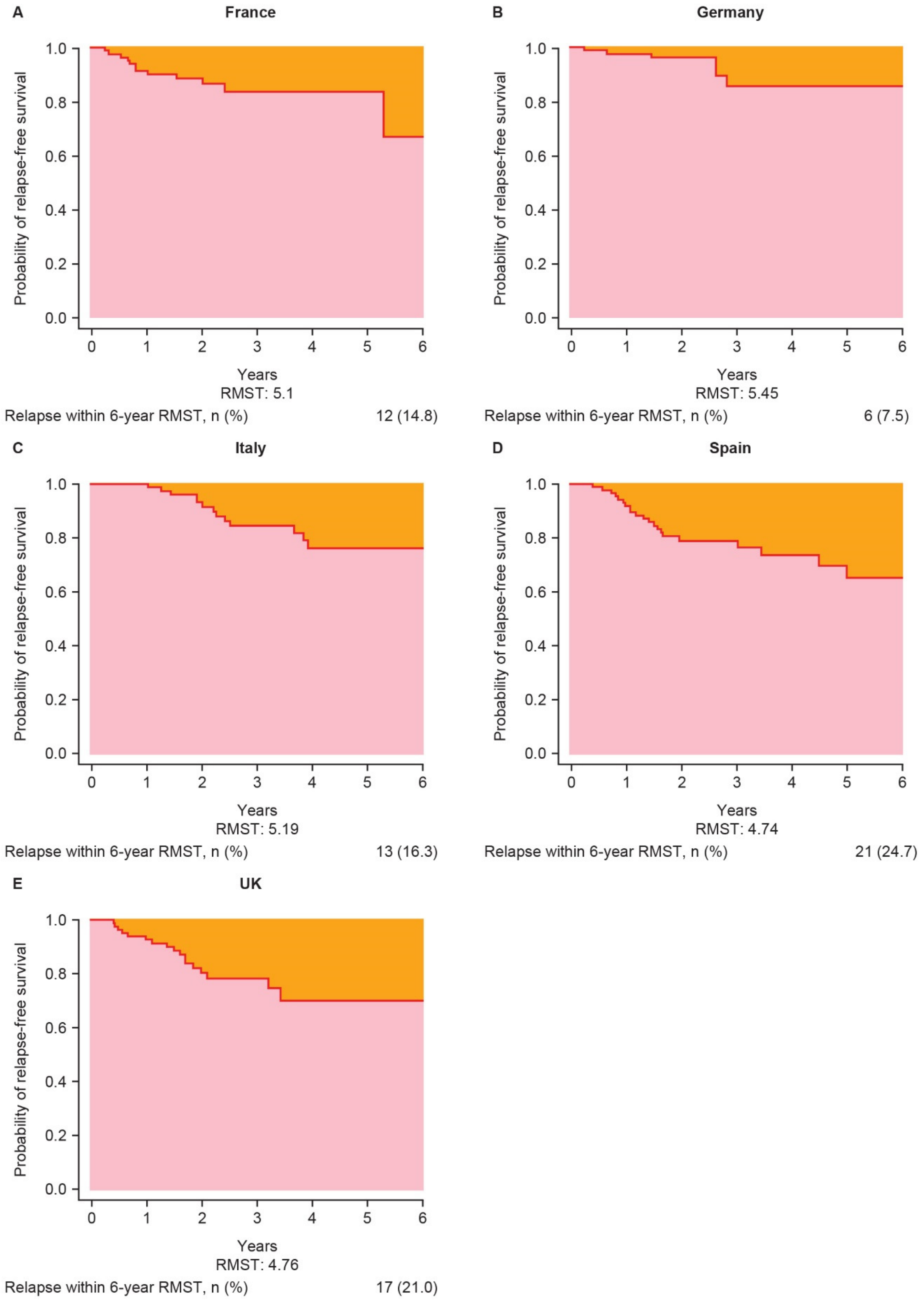

Figure S4. Relapse-free survival for patients with EGPA by country (A-E). EGPA, eosinophilic granulomatosis with polyangiitis; RMST, restricted mean survival time; UK, United Kingdom.

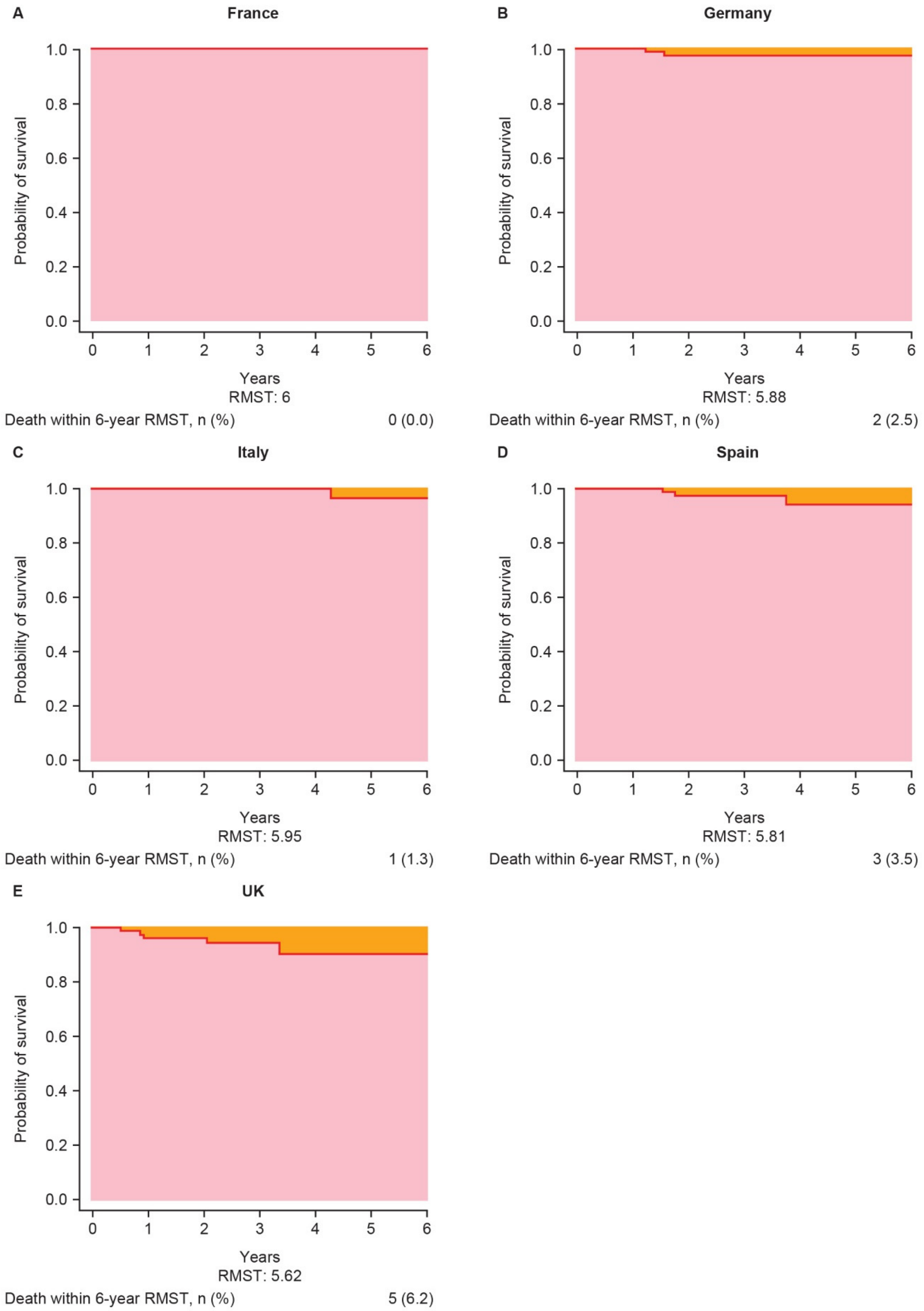

Figure S5. Overall survival in patients with EGPA by country (A-E). EGPA, eosinophilic granulomatosis with polyangiitis; RMST; restricted mean survival time; UK, United Kingdom**.**
